# mRNA decapping proteins regulate EIN2-dependent ethylene signaling in arabidopsis

**DOI:** 10.3389/fpls.2026.1897707

**Published:** 2026-06-30

**Authors:** Bai Hui Jiang, Yang Xie, Ai Ning Li, Jing Ying Yan, Gui Xin Li, Ji Ming Xu, Wo Na Ding, Dong Zhang, Zhe Ji, Chong Wei Jin, Nicholas P. Harberd, Shao Jian Zheng, Zhong Jie Ding, Eric J. Belfield, Meng Qi Cui

**Affiliations:** 1 State Key Laboratory of Plant Environmental Resilience, College of Life Sciences, Zhejiang University, Hangzhou, China; 2 Agricultural Experimental Station, Zhejiang University, Hangzhou, China; 3 College of Agronomy and Biotechnology, Zhejiang University, Hangzhou, China; 4 Ningbo Key Laboratory of Agricultural Germplasm Resources Mining and Environmental Regulation, College of Science and Technology, Ningbo University, Ningbo, China; 5 Institute of Quantitative Biology, College of Life Sciences, and School of Physics, Zhejiang University, Hangzhou, China; 6 Department of Biology, University of Oxford, Oxford, United Kingdom

**Keywords:** CTR1, DCP, P-body, signal transduction, translational repression

## Abstract

Ethylene signaling regulates plant growth and stress adaptation through a well-defined pathway, yet its mechanistic complexity remains underexplored. Here, we identify two mRNA decapping proteins, DCP1 and DCP2, as novel regulators of canonical ethylene signaling. Using forward genetics in the *ctr1–1* loss-of-function mutant (constitutively activated ethylene signaling), we identified *dcp1* and *dcp2* suppressor mutations that significantly rescue the developmental defects characteristic of *ctr1-1*. Transcriptome profiling showed that DCP1/2 regulates a substantial subset of genes that are affected by CTR1 or ACC treatment. We further found that both DCP1 and DCP2 were associated with the C-terminal of central regulator EIN2 (EIN2-C) in processing bodies (P-bodies). These *dcp* mutants exhibit reduced protein accumulation of the key transcription factor EIN3, suggesting a potential link to the EIN2-C-mediated translational regulation of EBF1/2 mRNAs. Additionally, we showed that the EMS-induced M78I mutation in DCP1 disrupts its interaction with DCP2, impairing DCP1-activated decapping activity of DCP2 *in vitro*, while the R138H mutation in DCP2 directly compromises both its enzymatic activity and the association with EIN2-C. Our findings provide genetic evidence that mRNA decapping machinery is involved in ethylene signaling and suggest a potential link to the EIN2-C-mediated pathway within P-bodies.

## Introduction

Ethylene is the only known gaseous phytohormone in plants. It plays pivotal roles in regulating plant growth, development, and responses to environmental stresses (Bleecker and Kende, 2000; Qiu et al., 2015; Hao et al., 2021; Wang et al., 2021; Huang et al., 2023; Masood et al., 2023). A foundational tool in ethylene signaling research has been the etiolated seedling ‘triple response’ phenotype, characterized by distinct morphological changes including a shortened and thickened hypocotyl and root, accompanied by an exaggerated apical hook curvature (Abeles et al., 1992). This phenotype has been instrumental in identifying core pathway components and elucidating their functions. Through molecular and genetic approaches, researchers have delineated a canonical ethylene signal transduction pathway (Guo and Ecker, 2004; Binder, 2020), while more intricate molecular regulatory networks continue to be refined.

Plant responses to ethylene originate from the perception of the hormone by ethylene receptors. In Arabidopsis, there are five receptors localized in the endoplasmic reticulum (ER) membrane (Binder, 2020). In the absence of ethylene, receptors such as ETHYLENE RESPONSE 1 (ETR1) form a complex with the negative regulator CONSTITUTIVE TRIPLE RESPONSE 1 (CTR1) (Kieber et al., 1993; Schaller and Bleecker, 1995; Clark et al., 1998). Within this complex, CTR1 phosphorylates ETHYLENE INSENSITIVE 2 (EIN2), preventing its cleavage and activation, thereby blocking downstream signal propagation (Ju et al., 2012; Qiao et al., 2012; Wen et al., 2012). Upon ethylene binding, conformational changes in the receptors inhibit CTR1 kinase activity (Alonso et al., 1999; Bisson and Groth, 2011). This inhibition stabilizes EIN2 and permits its C-terminal domain (EIN2-C) to be cleaved from the ER membrane, and the liberated EIN2-C in turn functions in both the cytosol and nucleus to transduce the ethylene signal (Qiao et al., 2009, Qiao et al., 2012; Wen et al., 2012; Li et al., 2015; Merchante et al., 2015).

Following its partial translocation into the nucleus, EIN2-C interacts with ENAP1 (EIN2 NUCLEAR ASSOCIATED PROTEIN 1) to regulate histone H3K14 and H3K23 acetylation. This chromatin remodeling facilitates ETHYLENE INSENSITIVE 3/EIN3-LIKE 1 (EIN3/EIL1)-mediated transcriptional activation of hundreds of downstream target genes, initiating the ethylene response (Wang et al., 2021; Zhang et al., 2016). On the other hand, a fraction of full-length EIN2 undergoes endoplasmic reticulum to the nucleus trafficking and proteolytic processing to generate functionally distinct EIN2-C isoforms that elicit downstream ethylene signaling outputs (Wen et al., 2012; Zhang et al., 2020). Furthermore, two independent studies demonstrated that cytosolic EIN2-C specifically binds the 3’ untranslated regions (3’UTRs) of *EBF1* and *EBF2* mRNAs encoding E3 ubiquitin ligases that target EIN3/EIL1 for degradation (Li et al., 2015; Merchante et al., 2015). Through association with P-body components (e.g., EIN5, PABs), EIN2-C sequesters these *EBF1/2* mRNAs into P-bodies, inhibiting their translation. This suppression stabilizes EIN3/EIL1 protein, enabling ethylene response activation independently of nuclear EIN3-mediated transcription Binder et al., 2004. In addition, ethylene-induced EIN3 accumulation triggers the transcriptional activation of EBF2 via direct binding to its specific promoter element (5’-TACAT-3’), initiating a negative feedback loop (Potuschak et al., 2006; Konishi and Yanagisawa, 2008; Chang et al., 2013). Consequently, the resulting EBF1/2 protein targets excess EIN3 for proteasomal degradation, preventing an exaggerated response to ethylene (Gagne et al., 2004). These findings demonstrate a non-transcriptional mechanism operating alongside the canonical pathway. However, given the multifaceted roles of EIN2 function (Zhang et al., 2020) and incomplete mechanistic understanding of P-body-mediated regulation, further detailed investigation into the precise regulatory mechanisms and interactions is warranted.

*CTR1*, encoding a Raf-like kinase (Kieber et al., 1993; Hua et al., 1995), acts upstream of EIN2 and is essential for suppressing the pathway in the absence of ethylene. The *ctr1–1* loss-of-function mutant exhibits a constitutive ethylene response phenotype, including severe growth stunting (Kieber et al., 1993; Negi et al., 2008; Mazzella et al., 2014; Park et al., 2023). This phenotype makes *ctr1–1* an ideal starting material for suppressor screens aimed at identifying novel pathway components. In this study, we performed an ethyl methanesulfonate (EMS)-based genetic suppressor screen on the *ctr1–1* mutant background. We isolated two suppressor mutations that significantly ameliorate the developmental defects associated with *ctr1-1*. These mutations reside in genes encoding mRNA decapping enzymes (DCP1 and DCP2) that regulate the expression of numerous ethylene-responsive genes. Notably, both proteins colocalize with EIN2-C in P-bodies, where they modulate EIN3 protein accumulation. These findings establish DCP1 and DCP2 as two novel regulators in EIN2-dependent ethylene signaling.

## Results

### Identification of novel components suppressing *ctr1–1* phenotypes​

**Figure 1:**
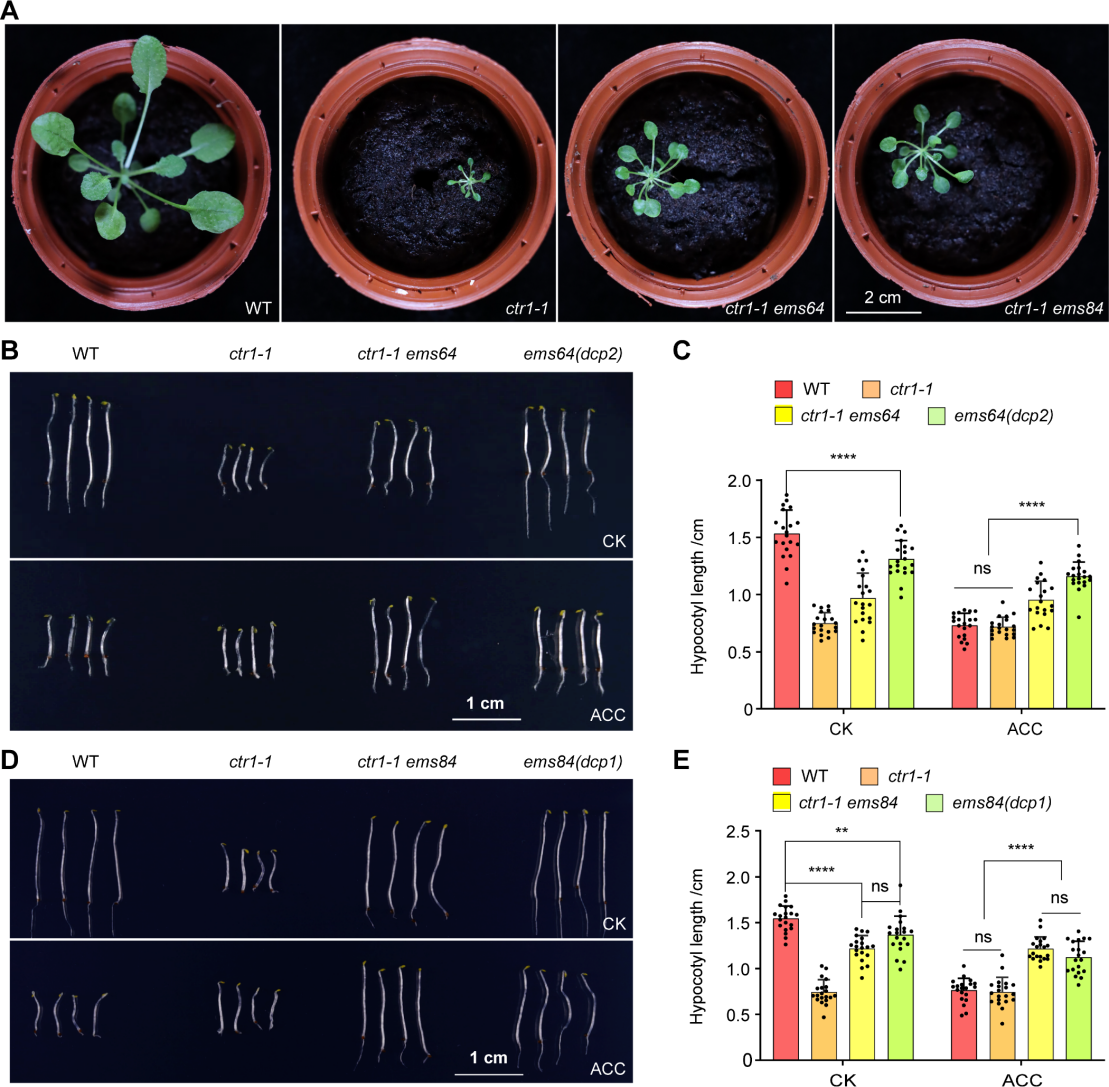
Phenotypes of WT, *ctr1-1*, *ctr1–1 ems64/84, ems64* (*dcp2*) and *ems84* (*dcp1*). **(A)** Phenotypes of *ctr1–1* and *ctr1–1 ems64/84* in soil culture. **(B)** Phenotypes and **(C)** hypocotyl length of *ctr1–1 ems64* and *ems64 (dcp2)* grown on ½ MS solid medium with or without 10 μM ACC treatment for 7 days under dark condition (n = 20). CK indicates control-check. **(D)** and **(E)** are the same cases in *ctr1–1 ems84* and *ems84* (*dcp1*). Data are shown as means ± SD. Data were analyzed by one-way ANOVA (n = 20) (‘ns’ means having no significant differences; **P < 0.01; ***P < 0.001; ****P < 0.0001).

To identify novel components involved in canonical ethylene signaling, we generated an ethyl methanesulfonate (EMS) mutagenesis library in the *ctr1–1* background. We screened for suppressor mutants that rescued the constitutive ethylene-response phenotype of *ctr1-1* (**Supplementary Figures 1A, B**). PCR amplification and Sanger sequencing revealed that the majority of suppressors contained mutations in *EIN2*, with additional mutations found in *EIN3* or *EIN5*, suggesting that this genetic screen is effective. Two suppressors without mutations in these known ethylene signaling genes, designated *ctr1–1 ems64* and *ctr1–1 ems84*, were selected for further characterization (**Figure 1A**).

We assessed ethylene responsiveness in *ctr1–1 ems64* and *ctr1–1 ems84* by performing the ethylene-specific triple response assay. Both mutants exhibited hypocotyl growth that was significantly less inhibited by the ethylene biosynthesis precursor 1-aminocyclopropane-1-carboxylic acid (ACC) compared to the *ctr1–1* parent or wild type (WT) (**Figures 1B–E**). This reduced sensitivity to ACC indicates that the suppressor mutations likely disrupt ethylene signal transduction downstream of the constitutive activation caused by *ctr1-1*. Consistent with this, the promoted root hair growth in *ctr1–1* was significantly attenuated in *ctr1–1 ems64* and *ctr1–1 ems84* (**Supplementary Figures 1C, D**).

### *DCP1* and *DCP2* are involved in ethylene response

To identify the causal mutations in *ctr1–1 ems64* and *ctr1–1 ems84*, we first backcrossed each mutant to *ctr1-1*. The F_1_ progeny exhibited phenotypes indistinguishable from *ctr1-1*, indicating that the suppressor mutations are recessive. Self-pollination of F_1_ plants yielded F_2_ populations exhibiting a 1:2:1 segregation ratio, with approximately one-quarter of plants displaying the respective *ctr1–1 ems* suppressor phenotype. This genetic segregation pattern indicates that a single recessive suppressor locus segregates in each mutant line.

**Figure 2:**
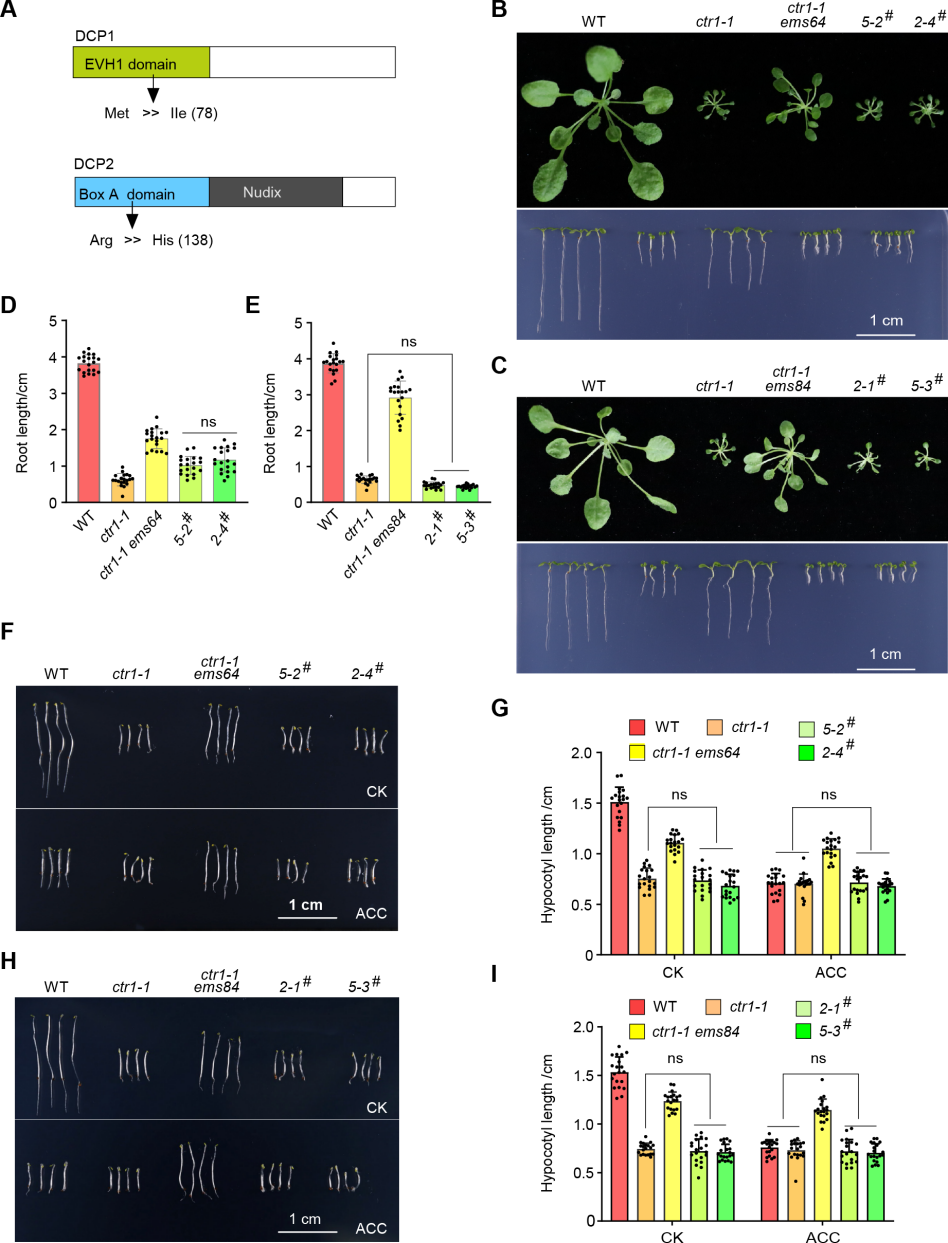
*DCP1* and *DCP2* restore the growth of *ctr1–1 ems84 and ctr1–1 ems64* mutants respectively. **(A)** Structure diagrams of DCP1and DCP2. **(B)** Growth of WT, *ctr1-1*, *ctr1–1 ems64*, and *pDCP2p::DCP2/ctr1–1 ems64* (5-2^#^ and 2-4^#^) on the soil (upper panel) and ½ MS solid medium (lower panel), respectively. **(C)** Growth of WT, *ctr1-1*, *ctr1-1ems84*, and *pDCP1p::DCP1/ctr1–1 ems84* (2-1^#^ and 5-3^#^) on the soil (upper panel) and ½ MS solid medium (lower panel), respectively. **(D)** Root length of indicated genotypes in the lower panel of Panel **(B)** (n = 20). **(E)** Root length of indicated genotypes in the lower panel of Panel **(C)** (n = 20). **(F, H)** Growth of indicated genotypes on ½ MS solid medium with or without 10 μM ACC treatment for 7 days under dark condition. **(G, I)** Hypocotyl length of the indicated genotypes in F and H, respectively (n = 20). Data are shown as means ± SD. Data in **(D, E)** were analyzed with one-way ANOVA, and data in **(G, I)** were analyzed with two-way ANOVA (‘ns’ means no significant differences; ****P < 0.0001).

We performed pooled whole-genome sequencing on suppressor-segregating F_2_ populations from both mutants. Analysis identified 12 and 6 ems-induced mutations within coding regions of *ctr1–1 ems64* and *ctr1–1 ems84*, respectively (**Supplementary Table 1**). Notably, both mutants harbored mutations in genes encoding core components of the mRNA decapping complex: *ctr1–1 ems64* contained a G-to-A mutation at nucleotide 795 of *DCP2* (At5g13570), resulting in an Arg138-to-His substitution (R138H) (**Figure 2A**); *ctr1–1 ems84* contained a G-to-A mutation at nucleotide 234 of *DCP1* (At1g08370), causing a Met78-to-Ile substitution (M78I) (**Figure 2A**). Both DCP1 and DCP2 are crucial components of the decapping complex, in which DCP1 interacts with DCP2 to alter its conformation, thereby enhancing its catalytic activity and improving its ability to decap mRNA (She et al., 2006, She et al., 2008). Given that decapping complexes comprising DCP1/2 are integral parts of P-bodies (Xu et al., 2006; Labno et al., 2016; She et al., 2004), which have been verified to participate in ethylene signaling (Li et al., 2015; Merchante et al., 2015), we hypothesized that the mutations in DCP1/2 may account for the phenotype of *ctr1–1 ems64* and *ctr1–1 ems84*.

Due to the lethal nature of homozygous *dcp1* and *dcp2* T-DNA insertion mutants (Xu et al., 2006) and the lack of significant difference in ethylene sensitivity between heterozygous mutants and WT, we introduced *DCP2* and *DCP1* genes (driven by their native promoters) into *ctr1–1 ems64* and *ctr1–1 ems84*, respectively, to determine whether *DCP1/2* can complement the phenotype of these suppressor mutants. We found that both *DCP2* and *DCP1* could indeed restore the development and ACC sensitivity of *ctr1–1 ems64* and *ctr1–1 ems84* to that of *ctr1-1*, respectively (**Figures 2B–I**). Furthermore, we crossed the *ctr1–1 ems64* and *ctr1–1 ems84* mutant to WT to remove the *ctr1–1* background but retain the mutations in *DCP2* and *DCP1*, generating *ems64* (hereinafter referred to as *dcp2*) and *ems84* (*dcp1*) mutants, respectively. We showed that both *dcp2* and *dcp1* exhibited reduced sensitivity to ACC treatment (**Figures 1B–E**). Notably, in the upper panel of **Figure 1B**, the root of *dcp2* is much longer than that of WT. We speculate that this abnormal root phenotype may be caused by one or more background EMS-induced mutations (**Supplementary Table 1**). These results collectively indicate that DCP1 and DCP2 are involved in the ethylene response, and that mutations at Met78 in DCP1 and Arg138 in DCP2 both disrupt this response.

To investigate the dosage effect of DCP proteins, we generated *35S:DCP1-GFP* and *35S:DCP2-GFP* overexpression lines (**Supplementary Figure 2A**). Under dark-grown conditions without ACC treatment, neither overexpression line exhibited a constitutive ethylene response phenotype. Upon ACC treatment, however, the *35S:DCP2-GFP* seedlings displayed a hypersensitive phenotype with significantly shorter hypocotyls compared to the WT, whereas the *35S:DCP1-GFP* seedlings showed ACC sensitivity similar to the WT (**Supplementary Figures 2B, C**). Given that DCP1 acts primarily as an auxiliary subunit to facilitate DCP2 activity (Beelman et al., 1996; Floor et al., 2010), overexpressing DCP1 alone may be insufficient to enhance signaling output without a corresponding increase in the catalytic subunit DCP2. The hypersensitivity in *DCP2*-overexpressing lines contrasts with the reduced sensitivity of the *dcp2* mutant, highlighting a positive correlation between DCP2 dosage and ethylene signaling output.

### DCP1 and DCP2 mediate ethylene signaling

**Figure 3:**
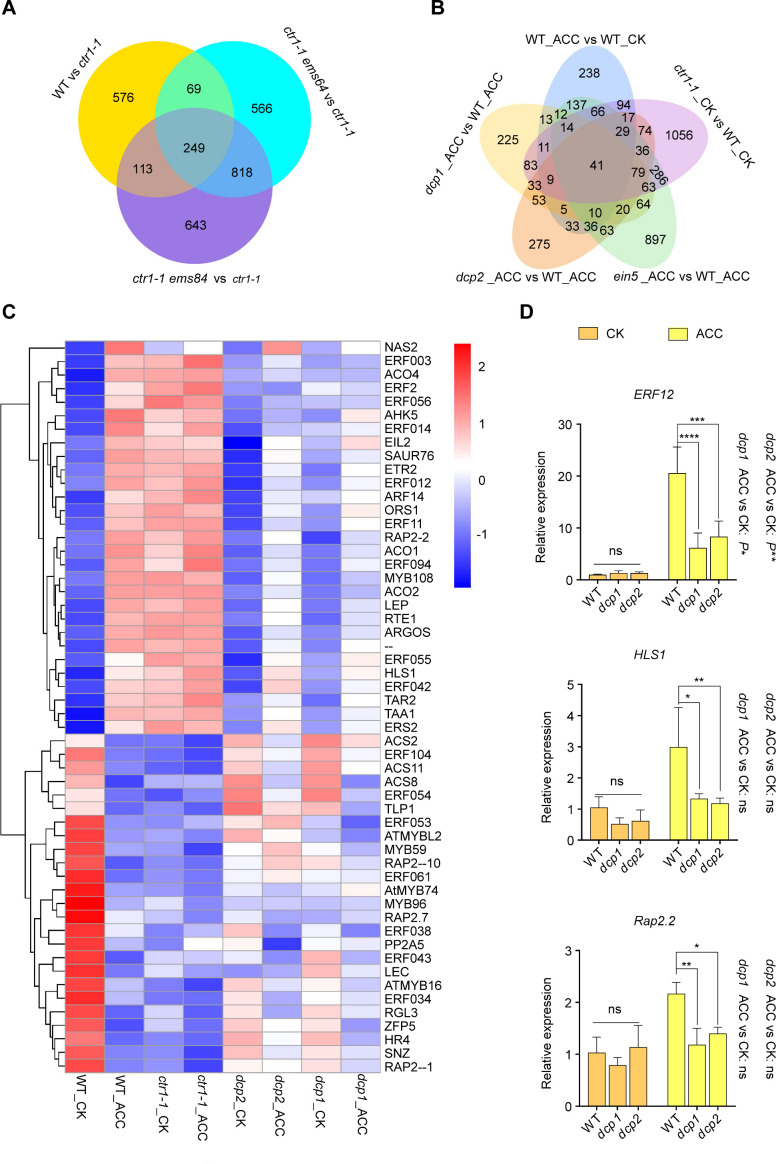
Transcriptome analysis revealing the involvement of DCP1/2 in ethylene signaling. **(A)** Venn diagram of differentially expressed genes (DEGs; fold change ≥ 2, *P* < 0.05) in dark-grown seedlings of WT vs *ctr1-1*, *ctr1–1 ems64* vs *ctr1-1*, and *ctr1–1 ems84* versus *ctr1–1* without ACC treatment. **(B)** Venn diagram of DEGs (fold change ≥ 2, *P* < 0.05) in dark-grown seedlings of WT_ACC vs WT_CK, *ctr1-1*_CK vs WT_CK, *dcp1/2*_ACC vs WT_ACC. **(C)** Clustering diagram of the expression profiles of ethylene-related genes that are differentially expressed (fold change ≥ 2, *P* < 0.05) in WT_ACC versus WT_CK among different lines. **(D)** Expression analysis of *ERF12*, *HLS1* and *Rap2.2* (n = 3). Data are shown as means ± SD. Data were analyzed with one-way ANOVA (‘ns’ means no significant differences, *P < 0.05, **P < 0.01, ***P < 0.001, ****P < 0.0001).

To investigate whether DCP1 and DCP2 regulate ethylene signaling transduction, we conducted transcriptomic analyses of WT, *ctr1-1*, *ctr1–1 ems84* (*dcp1* in *ctr1-1*), and *ctr1–1 ems64* (*dcp2* in *ctr1-1*) dark-grown seedlings without exogenous ACC. The constitutively activated ethylene signaling in *ctr1–1* significantly altered the expression of 1007 genes compared to WT (**Figure 3A**; **Supplementary Dataset 1**). Notably, approximately 32% (318 genes) and 36% (362 genes) of these differentially expressed genes (DEGs) were normalized (*i.e.*, suppressed towards WT levels) in the *ctr1–1 dcp1* and *ctr1–1 dcp2* backgrounds, respectively (**Figure 3A**), indicating that DCP1 and DCP2 mediate a substantial proportion of CTR1-dependent ethylene signal transduction.

To identify ethylene-dependent DEGs specifically influenced by DCP1/DCP2, we compared the transcriptomes of ACC-treated dark-grown seedlings of WT, *dcp1*, and *dcp2*. Relative to ACC-treated WT, we identified 735 and 813 DEGs in *dcp1* and *dcp2* mutants, respectively (**Figure 3B**; **Supplementary Dataset 2**). Given that EIN5 (XRN4) functions as the downstream 5’–3’ exonuclease following decapping and is a known regulator of ethylene signaling (Li et al., 2015; Olmedo et al., 2006; Souret et al., 2004), we reasoned that if DCP1/2 act within this mRNA decay pathway, their transcriptomic profiles should exhibit significant overlap with *ein5*. To test this functional convergence, we included *ein5* mutants in the RNA-seq analysis. This analysis identified 1853 DEGs in *ein5* under ACC treatment. Notably, substantial overlaps were observed: 51% (373 DEGs) in *dcp1*, 49% (402 DEGs) in *dcp2*, and 44% (809 DEGs) in *ein5* were also transcriptionally regulated by ACC in WT or constitutively altered in *ctr1-1* (**Figure 3B**), suggesting a coregulatory relationship. Heatmap analysis confirmed that the expression of established ethylene-responsive genes was similarly attenuated in both *dcp1* and *dcp2* mutants under ACC treatment (**Figure 3C**). Subsequent validation through qPCR of three randomly selected genes confirmed the regulatory roles of DCP1/2 in ethylene-responsive gene expression, and also verified the reliability of transcriptomic sequencing data (**Figure 3D**). Collectively, these findings demonstrate that DCP1/2 play pivotal roles in ethylene signaling transduction through their extensive modulation of ethylene-responsive transcriptome networks.

### DCP1 and DCP2 associate with EIN2-C to regulate EIN3 accumulation

**Figure 4:**
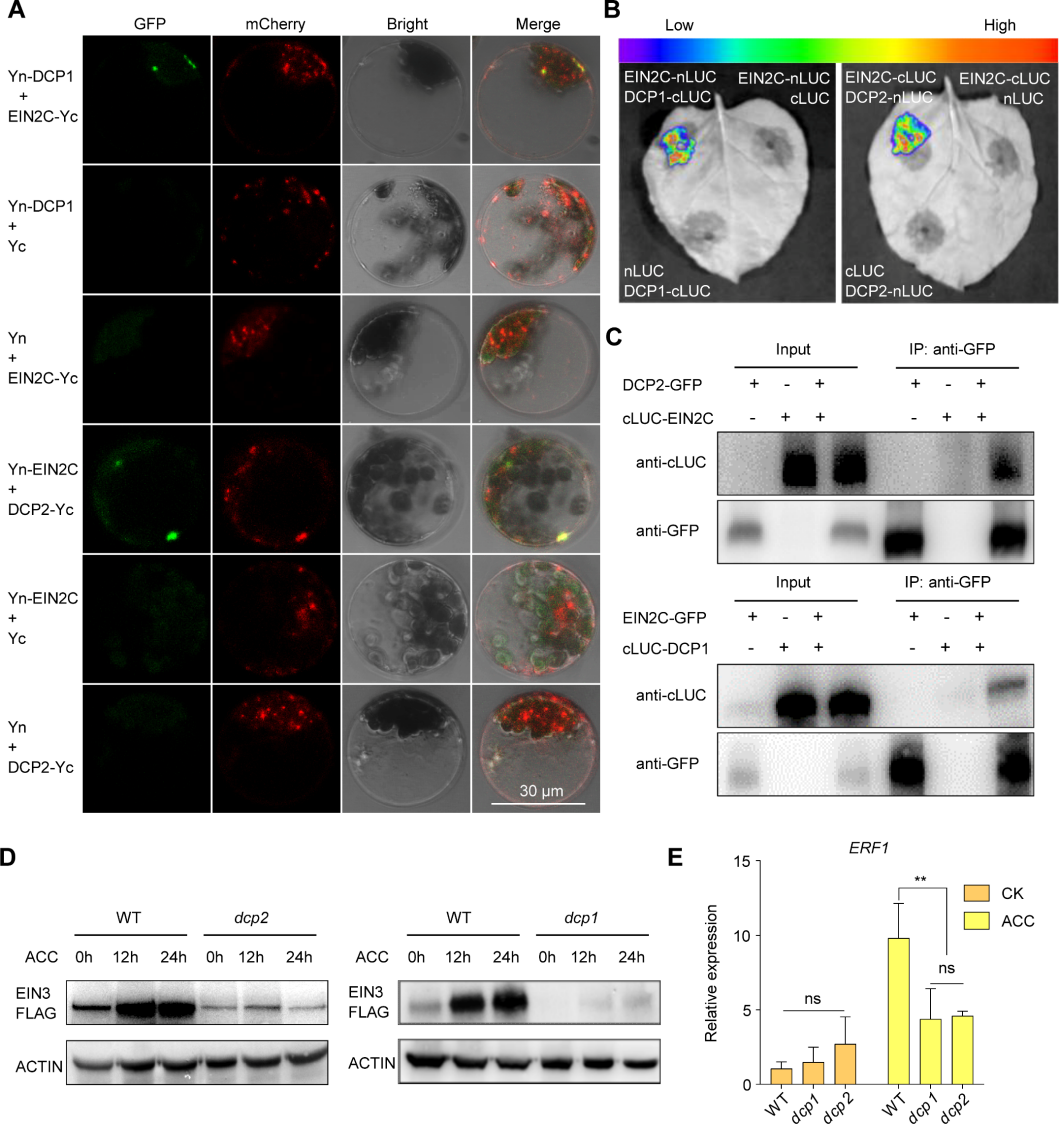
Interaction between DCP1/2 and EIN2-C. **(A)** BiFC and **(B)** Split-LUC assays showing the interaction of DCP1and DCP2 with EIN2-C. DCP5-mCherry was used as a P-body marker in **(A)**. **(C)** Interaction between EIN2C and DCP1/2 revealed by Co-immunoprecipitation (Co-IP) assays. **(D)** Immunoblot analysis of EIN3 protein levels in WT, *dcp1* and *dcp2* with time course of ACC treatment. Total proteins were extracted from whole plants. EIN3 protein levels were detected with the anti-Flag antibody. Detection of ACTIN in each immunoblot analysis was used as the loading control. **(E)** Expression analysis of *ERF1* in WT, *dcp1* and *dcp2* under control and ACC treatment. Total RNAs were extracted from 7-day-old seedlings under control and ACC treatment for 4.5 hrs (n = 3). Data are shown as means ± SD. Data were analyzed with two-way ANOVA (‘ns’ means no significant differences, **P < 0.01).

We next investigated the mechanism by which DCP1/DCP2 influence ethylene signaling. We found that while ACC treatment did not affect the mRNA levels of DCP1/2 (**Supplementary Figure 3**), it significantly induced the protein accumulation of DCP1 (**Supplementary Figure 4**; we failed to detect GFP fluorescence in the *DCP2-GFP* transgenic lines), suggesting that DCP1 is likely regulated by ethylene at post-translational level. Since DCP1 and DCP2 are core components of P-bodies, which serve as critical platforms for EIN2-mediated translational repression of *EBF1/2* during ethylene signaling (Li et al., 2015; Merchante et al., 2015), we hypothesized that DCP1/2 might regulate EBF1/2 translation through association with the C-terminal domain of EIN2 (EIN2-C). Bimolecular fluorescence complementation (BiFC) assays confirmed the association between DCP1/2 and EIN2-C, with colocalization observed in cytoplasmic puncta consistent with P-bodies (**Figure 4A**; DCP5-mCherry was used as a P-body marker). This interaction was further corroborated by split-luciferase (Split-LUC) complementation assays and Co-immunoprecipitation (Co-IP) assays (**Figures 4B, C**). These findings demonstrate that DCP1/2 likely participate in EIN2-dependent ethylene signal transduction. Moreover, the convergent transcriptomic profiles of *dcp* and *ein5* mutants prompted us to examine whether DCP1/2 physically interact with EIN5 to form a functional mRNA decay module. BiFC assays confirmed that DCP1 and DCP2 associate with EIN5 (**Supplementary Figure 5**). Notably, *dcp1* and *dcp2* mutations did not disrupt this interaction (**Supplementary Figure 6**), indicating that the ethylene-related phenotypes are not attributable to a loss of EIN5 binding, ruling out this indirect mechanism and supporting a direct role for DCP1/DCP2 in EIN2-mediated translational regulation.

We next examined the mRNA levels of *EIN2* and *EBF1/2* in the *dcp1* and *dcp2* mutants to determine whether the observed phenotypes resulted from transcriptional feedback or altered mRNA stability. The results showed that the mRNA levels of *EBF1/2* were comparable to those in the WT, and the expression of *EIN2* was not significantly changed versus WT under ACC treatment (**Supplementary Figures 7**, **8**). Attempts to assess EBF1/2 translation efficiency directly in these genotypes using translating ribosome affinity purification coupled with qPCR (TRAP-qPCR) were unsuccessful due to extremely low levels of *EBF1/2* mRNA (<1 transcript per cell equivalent) captured on ribosomes across all genotypes (Ct values > 35). Nevertheless, given that EBF1/2 abundance directly regulates EIN3/EILs protein stability (Guo and Ecker, 2004; Zhang et al., 2017; Yan et al., 2024), we instead examined EIN3 protein levels in WT and *dcp1/2* mutants. Intriguingly, ACC-treated mutants exhibited markedly reduced EIN3 protein accumulation compared to WT (**Figure 4D**), whereas they showed comparable EIN3 transcriptional levels (**Supplementary Figure 9**). Consistent with this observation, transcript levels of *ERF1*—a direct downstream target of EIN3 (Chao et al., 1997)—were significantly decreased in the mutants versus WT (**Figure 4E**). Collectively, these results suggest that DCP1/2 indirectly modulate EIN3 protein accumulation, thereby regulating ethylene signaling output.

### The EMS-induced mutations in DCP1/2 affect DCP function

**Figure 5:**
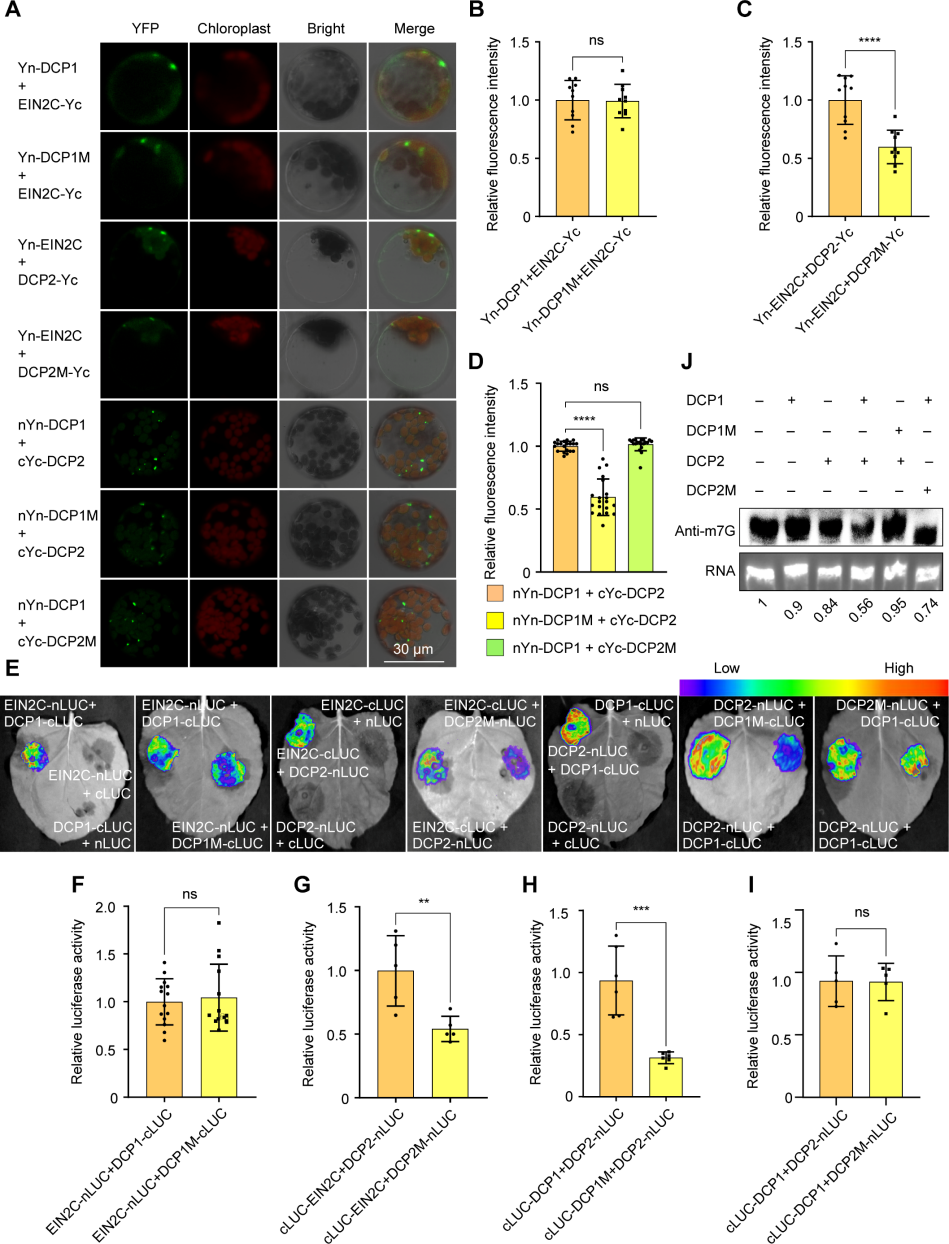
EMS-induced mutations in DCP1/2 affect DCP function. **(A)** BiFC and **(E)** Split-LUC analysis showing the EMS-induced mutations in DCP1/2 affect DCP2-EIN2 and DCP1-DCP2 interaction. ‘DCP1M’ means DCP1 with M78I mutation, ‘DCP2M’ means DCP2 with R138H mutation. **(B–D)** The fluorescence intensity of BiFC in **(A, F–I)** The luciferase intensity of LUC in **(E, J)** *In vitro* decapping assays showing the EMS-induced mutations in DCP1/2 affect DCP decapping function. The mRNA used for the experiment was the EZ Cap™ Firefly Luciferase mRNA. The decapping activity of DCPs was assessed by monitoring the reduction of 5’-cap structures in mRNAs. The cap structure was detected with Anti-7-Methylguanosine antibody. Detection of RNA in each analysis was used as the loading control. Data are shown as means ± SD. Data were analyzed with one-way ANOVA and unpaired t test (‘ns’ means no significant differences; **P < 0.01, ***P < 0.001, ****P < 0.0001.

Since the EMS-induced mutations lead to M78-to-I substitution in DCP1 and R138-to-H in DCP2 (**Figure 2A**), we next investigated how these mutations affect ethylene signaling. We first determined whether they affect the interaction between DCP1/2 and EIN2-C. The BiFC and Split-LUC assays demonstrated that mutation of DCP2 substantially attenuated its association with EIN2-C, while mutation of DCP1 did not (**Figures 5A–C, E–G**), suggesting that the R138-to-H mutation of DCP2 likely compromises its recruitment by EIN2-C in P-bodies, and therefore reduces ethylene signaling. Since DCP1 acts as an activator to interact with DCP2 and enhance its catalytic activity (She et al., 2006, She et al., 2008), we then compared the effect of EMS-induced mutations on DCP1-DCP2 interaction. We showed that the M78 mutation of DCP1 notably inhibited its interaction with DCP2 (**Figures 5A, D, E, H**), whereas the R138 mutation of DCP2 did not (**Figure 5A, D, E, I**). This indicates that the DCP1 M78I mutation prevents the activation of DCP2 by disrupting complex formation. To exclude potential confounding effects of other factors on protein function, we performed Western blot analysis on the samples from the luciferase assay shown in **Figure 5E**. The results demonstrated that all proteins were fully expressed at expected levels (**Supplementary Figures 10**–**13**).

To further verify if the EMS-induced mutation of DCP1/2 affect the mRNA decapping activity of DCP2, we conducted an *in vitro* decapping experiment using recombinant DCP1/2 proteins and synthesized capped *LUC* mRNAs. The decapping activity can be reflected by the abundance of m7G in the mRNAs, and lower abundance indicates higher decapping activity. We showed that while DCP2 alone had low decapping activity, the presence of DCP1 obviously enhanced DCP2 activity (**Figure 5J**). In the single-addition controls, the DCP2M (R138H) protein alone exhibited decapping activity comparable to wild-type DCP2 (**Supplementary Figure 14**). We attribute this result to the extremely low basal activity of DCP2 in the absence of DCP1, which makes it difficult to detect subtle differences between the WT and mutant proteins under these conditions. Nevertheless, the stimulatory effect typically provided by WT DCP1 was severely compromised when paired with DCP2M (**Figure 5J**), indicating that the R138H mutation impairs the catalytic efficiency of DCP2 or its responsiveness to activation. Conversely, the DCP1M (M78I) protein showed a reduced ability to enhance WT DCP2 activity (**Figure 5J**), consistent with its weakened interaction with DCP2 (**Figure 5H**). Taken together, these results indicate that the EMS-induced mutations in DCP1 and DCP2 attenuate ethylene signaling through either repression of DCP2 decapping activity or disconnection between EIN2-C and RNA-decay machinery.

## Discussion

Forward genetics has played a pivotal role in elucidating the ethylene signaling pathway in plants (Wen, 2015). Key pathway components, including receptors like ETR1, the kinase CTR1, the central regulator EIN2 and the key transcription factor EIN3 were originally identified through classical, wild-type-based forward genetic screens (Bleecker et al., 1988; Chang et al., 1993; Kieber et al., 1993; Chao et al., 1997; Alonso et al., 1999; Bisson and Groth, 2010). However, this conventional approach has yielded diminishing returns for discovering novel, functionally significant genes. This limitation likely arises from following factors: ​​(i)​​ functional redundancy within the pathway, where gene ablation fails to produce a discernible phenotype, ​​(ii)​​ embryonic or early lethality associated with the loss of certain essential genes, and (iii) the insufficient sensitivity of traditional screens to reliably detect mutants exhibiting weaker ethylene insensitivity. To overcome these barriers and uncover previously hidden genetic regulators, we employed a suppressor mutagenesis screen utilizing the gain-of-function *ctr1–1* mutant. This mutant displays a pronounced, easily scorable dwarf phenotype due to constitutive ethylene response signaling. Screening for suppressors that restore tall growth provides a sensitive phenotypic readout (suppression of dwarfism​​) and a strong selective advantage for identifying mutations compensating for *ctr1–1* hyperactivation.

**Figure 6:**
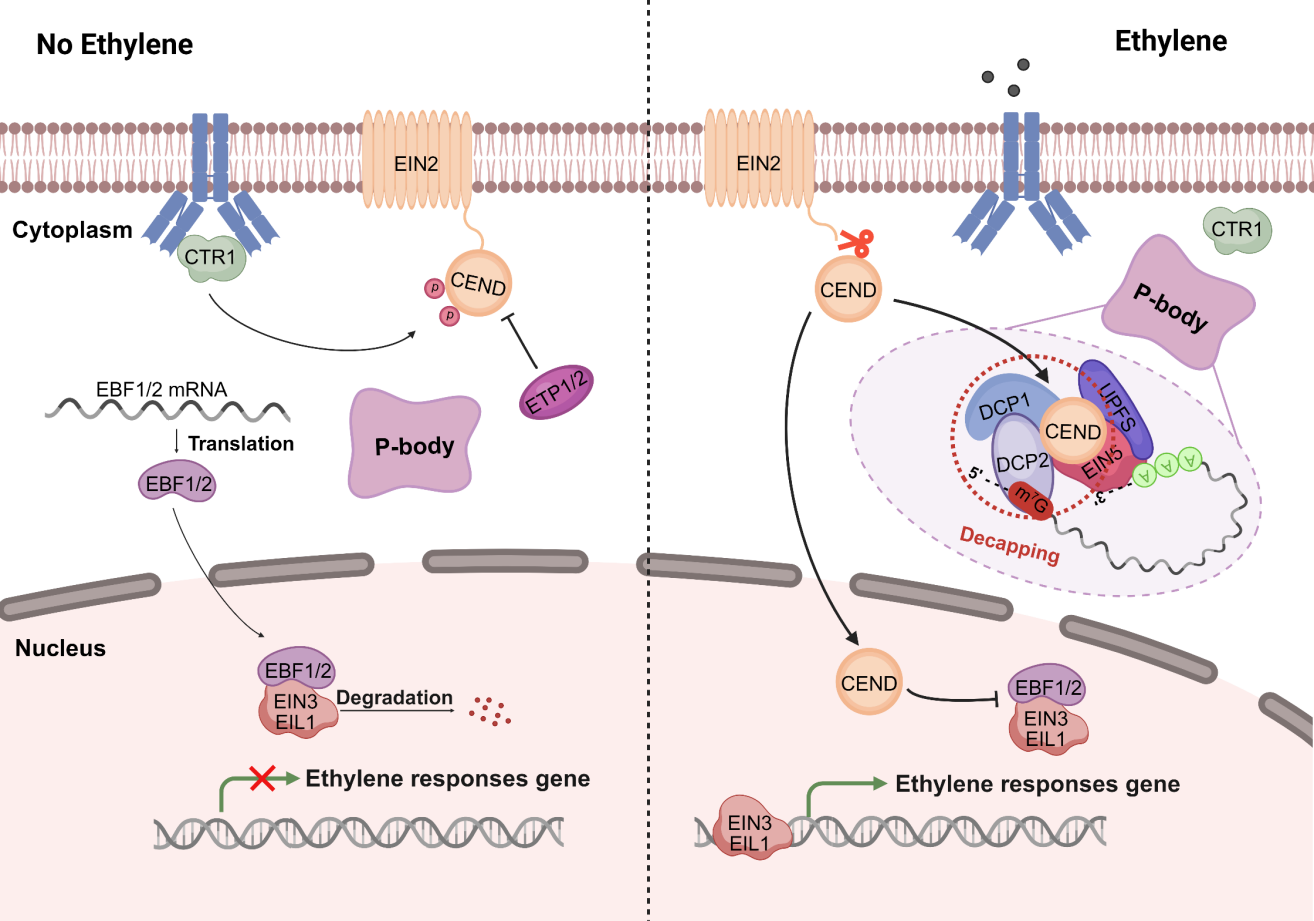
Schematic diagram of DCP1/2 participating in ethylene signaling. In the absence of ethylene, receptors such as ETR1 form a receptor complex with CTR1 and enhance its kinase activity. CTR1 promotes the degradation of EIN2 via an ETP protein-mediated ubiquitination pathway by phosphorylating EIN2. When ethylene is present, the function of CTR1 is inhibited, enabling the cleavage of the C terminal of EIN2 (CEND). A portion of EIN2-C enters the nucleus to participate in ethylene signal transduction, while another portion mediates translational repression of EBF1/2 within P-bodies. The decapping enzymes in P-bodies are recruited by EIN2-C, with DCP2 participating in the decapping reaction and DCP1 interacting with DCP2 to enhance its decapping activity.

This study presents a suppressor screen using the *ctr1–1* mutant, identifying DCP1 and DCP2 as novel regulators of ethylene signaling. Our analyses reveal that these proteins interact directly with the C-terminal domain of EIN2. Through this interaction, DCP1/DCP2 likely mediate translational suppression of *EBF1/2* mRNA within processing bodies (P-bodies). This suppression may reduce EBF1/2 protein levels, leading to diminished degradation of the master transcription factor EIN3 and consequent accumulation of EIN3 protein to drive ethylene responses (**Figure 6**).​ These findings offer novel mechanistic insights into the ethylene signaling cascade,​​ uncovering a previously unrecognized layer of post-transcriptional control involving DCP1, DCP2, and P-body-mediated translational regulation that operates downstream of EIN2 to fine-tune EIN3 stability and output.

In eukaryotic systems, mRNA translation and degradation serve as critical regulatory checkpoints for protein homeostasis, with these processes being mechanistically interconnected (Bicknell and Ricci, 2017; Decker and Parker, 1993). The degradation cascade involves three essential steps: deadenylation, decapping, and exonucleolytic digestion. Of these, decapping, the enzymatic removal of the m7G cap structure, represents a rate-limiting step that irreversibly commits mRNAs to degradation while simultaneously terminating translation (Franks and Lykke-Andersen, 2008; Houseley and Tollervey, 2009). The mRNA decapping involves the formation of a complex by DCP2, DCP1, and VARICOSE (VCS) in P-bodies, which acts synergistically in the regulation of mRNA storage and degradation (Xu et al., 2006). DCP2 has been demonstrated as the active component for m^7^G-CAP hydrolysis (Gunawardana et al., 2008), playing a central role by catalyzing the hydrolysis of the mRNA 5’ end (Labno et al., 2016). In contrast, DCP1 functions as a major auxiliary subunit that facilitates the catalytic activity of DCP2 (Beelman et al., 1996; Floor et al., 2010). Notably, our data reveal a previously unrecognized role for DCP1/2 in ethylene signaling. While *DCP1/2* mutations did not alter *EBF1/2* mRNA abundance (**Supplementary Figure 7**), both mutations significantly reduced EIN3 protein accumulation and *ERF1* expression (**Figures 4D, E**). Since EBF1/2 directly control the protein stability of EIN3 (Guo and Ecker, 2003; Potuschak et al., 2006), this observation aligns with established models where EIN2-C sequesters *EBF1/2* mRNA in P-bodies to inhibit their translation (Li et al., 2015; Merchante et al., 2015). Considering that DCP1/2 are important participants in the decapping process preceding mRNA degradation and interact with EIN2-C (**Figure 4**), we propose a model in which EIN2-C recruits DCP1/2 to modulate translational repression of *EBF1/2* mRNAs. However, we acknowledge that direct evidence linking DCP1/2 to the specific modulation of *EBF* mRNA translation is currently lacking. While we cannot exclude alternative mechanisms (e.g. indirect effects on translation, regulation of other unknown factors, or general alterations in P-body integrity), the specific interaction between DCP1/2 and EIN2-C, combined with the selective reduction of EIN3 protein accumulation in the mutants, supports the plausibility of this recruitment model. Further investigation (e.g. utilizing emerging sensitive ribo-seq techniques) is required to definitively establish the direct impact on *EBF1/2* translation efficiency. Nevertheless, this work bridges the ethylene signaling pathway with the fundamental mRNA decapping machinery, suggesting a novel layer of post-transcriptional control. The transcriptomic convergence with *ein5* and the maintained physical interaction with the downstream exonuclease EIN5 further confirm that DCP1/2 operate within the canonical mRNA decay pathway (**Figure 3B**; **Supplementary Figure S5**); notably, the intact DCP-EIN5 association in the mutants supports the model that their signaling defects stem from specific catalytic or recruitment impairments rather than a global disruption of the degradation machinery. These findings additionally suggest that the mRNA decapping machinery is not only required for mRNA decay process, but also likely for mRNA translational repression.

Structural insights may provide further mechanistic clarity. DCP2, which belongs to the Nudix hydrolase family, contains a Nudix domain and an N-terminal regulatory domain (NTD) (Xu et al., 2006; She et al., 2008). The NTD interacts with DCP1 and is essential for DCP2’s decapping activity, likely by optimizing enzymatic efficiency (She et al., 2008). In this study, the DCP2 R138H mutation, which occurred precisely in the NTD (**Figure 2A**), did not disrupt the DCP1-DCP2 interaction (**Figures 5A, D, E, I**), but remarkably impaired DCP2 decapping activity (**Figure 5J**), indicating a critical role for the NTD in intrinsic catalytic activity beyond mediating DCP1 binding. This impairment may arise from disruption of an intramolecular interaction between the NTD and the catalytic Nudix domain (Mildvan et al., 2005; She et al., 2006; Gunawardana et al., 2008). Moreover, the R138H mutation abolished the association between DCP2 and EIN2-C (**Figures 5A, C, E, G**), suggesting that the NTD is also necessary for DCP2 recruitment by EIN2-C. Regarding DCP1, its EVH1 domain binds the DCP2 NTD. This interaction induces a catalytically active conformation in DCP2, likely by positioning the Nudix domain for substrate engagement (She et al., 2006, She et al., 2008). The M78I mutation resides within the DCP1 EVH1 domain (**Figure 2A**). While this mutation did not affect DCP1’s interaction with EIN2-C, it specifically impaired binding to DCP2 (**Figure 5**). Combined with the decapping defect observed in the DCP2 R138H mutant (**Figure 5J**), this supports the model that DCP1 participation in ethylene signaling occurs primarily by enhancing DCP2’s decapping activity. Specifically, this enhancement is likely required for efficient EIN2-C-mediated suppression of EBF1/2 mRNA translation, which in turn regulates EIN3 protein accumulation (**Figure 4D**). Notably, the distinct phenotypes of overexpression lines (**Supplementary Figure 2**) further support the functional divergence between DCP1 and DCP2. The hypersensitivity of DCP2-overexpressing lines aligns with its catalytic role; an abundance of DCP2 likely facilitates more efficient recruitment by EIN2-C, amplifying the ethylene response. In contrast, DCP1 functions as an auxiliary subunit, therefore overexpressing it alone is insufficient to alter signaling output if the catalytic engine (DCP2) or the recruitment signal (EIN2-C) is limiting.

Since DCP1 and DCP2 are core components of the general mRNA decay machinery, a critical question is how specificity is achieved in ethylene signaling. We propose that this specificity is not intrinsic to DCP1/2 but is mediated by recruitment via the pathway-specific regulator EIN2-C. In this model, EIN2-C acts as a molecular adaptor that binds both the 3’ UTR of *EBF1/2* mRNAs and the decapping complex, thereby recruiting DCP1/2 specifically to ethylene-responsive transcripts. This mechanism is conceptually similar to the function of MHZ9 in rice (Huang et al., 2023). Both pathways utilize EIN2 to target *EBF* mRNAs, but the molecular executors differ: rice employs the specific RNA-binding protein MHZ9 to recognize *EBF* transcripts, whereas Arabidopsis recruits the core decapping machinery (DCP1/2) via EIN2-C. This highlights an evolutionary theme where the conserved ethylene signaling module (EIN2-EBF-EIN3) adapts distinct RNA regulatory factors, lineage-specific adaptors or repurposed core decay enzymes, to achieve specific hormonal control. Consistent with this adaptor-mediated model, the *dcp2* (R138H) allele specifically disrupts the interaction with EIN2-C and ethylene sensitivity while retaining basal decapping activity sufficient for viability, demonstrating that the signaling function can be genetically separated from the general housekeeping role.

Given that numerous ethylene signaling components (e.g., EIN2, EIN3, EIN5) were identified through forward genetic screens, it raises the question of why DCP1 and DCP2 remained undetected in such approaches. We propose the following explanations: First, homozygous mutations in DCP1/2 can be lethal to plants (Xu et al., 2006). Second, functional mutations in DCP1/2 may need to confer a hypomorphic effect to be phenotypically scorable; such mutations would need to impair decapping activity sufficiently to produce a discernible ethylene signaling phenotype, yet not so severely as to compromise plant viability or essential developmental processes. Supporting the plausibility of this scenario, Thran et al. (2012) identified a mutation at the identical R138 residue in DCP2 (as studied here). This mutation reduced substrate binding affinity and decapping activity, impairing transgene silencing without causing lethality, demonstrating that specific mutations can modulate DCP2 function without catastrophic consequences. Additionally, given the essential role of DCP1/2 in the regulation of mRNA homeostasis, EMS-induced mutations in *DCP1* and *DCP2* inevitably impact plant growth, with both *dcp1* and *dcp2* mutants showing to some extent compromised growth versus WT under normal condition.

In summary, this study uncovers ​​DCP1 and DCP2, core components of the mRNA decapping machinery and P-bodies, as essential novel regulators within the canonical ethylene signaling pathway.​​ Given that mRNA decapping by the DCP complex is the critical, ​​rate-limiting step​​ initiating translational silencing and mRNA degradation within P-bodies (Franks and Lykke-Andersen, 2008), our findings ​​provide a model for the previously established role of P-bodies and EIN2-C in repressing *EBF1/2* mRNA translation​​ (Li et al., 2015; Merchante et al., 2015). The discoveries of mRNA decapping as a regulated step in plant hormone signaling would open new avenues for exploring how RNA fate regulation broadly interfaces with hormone response networks in plants.

## Materials and methods

### Plant materials and growth conditions

The genotypes of plants used in this study include the Wild-Type (WT) Columbia-0 (Col-0) and the *ctr1–1* mutant. The *ctr1–1* mutant carries a T-to-A point mutation at nucleotide position 4025 within the *CTR1* gene, leading to an amino acid substitution of aspartic acid (Asp) to glutamic acid (Glu) at residue 694, as previously described by Kieber et al. (1993). Additionally, two EMS-induced mutant derivatives of *ctr1-1*, designated *ctr1–1 ems84* and *ctr1–1 ems64*, were also included in this study. Two complementation lines were generated as follows. For the *pDCP1p::DCP1*/*ems84* × *ctr1–1* line, full-length *DCP1* driven by its native promoter was introduced into *ems84*, followed by crossing with *ctr1-1*. Homozygous lines were confirmed via PCR (primers in **Supplementary Data**). For the *pDCP2p::DCP2*/*ctr1–1 ems64*, full-length *DCP2* driven by its native promoter was directly transformed into *ctr1-1ems64*.

Overexpression lines were generated by introducing the *35S::EIN3-3×FLAG* into different genetic backgrounds. Specifically, transgenic lines expressing EIN3 fused to a triple FLAG tag (EIN3-3×FLAG) under the control of the 35S promoter were created in WT, *ems84*, and *ems64* backgrounds, designated as *35S::EIN3-3×FLAG/WT*, *35S::EIN3-3×FLAG/ems84*, and *35S::EIN3-3×FLAG/ems64*, respectively. To achieve this, the full-length coding sequence of *EIN3* was cloned into the *pCAMBIA1300-35S-3×FLAG* vector, resulting in the *35S::EIN3-3×FLAG* plasmid. This T-DNA was then transformed into WT, *ems84*, and *ems64* plants using *Agrobacterium tumefaciens* strain *GV3101*.

Plants were grown in an environmental chamber maintained at a 16-h light/8-h dark cycle with temperatures set at 23 °C during the day and 21 °C at night. Seeds were surface-sterilized by immersion in 75% ethanol, followed by three rinses with sterile deionized water, and subsequently sown onto 1/2 Murashige and Skoog (MS) solid medium adjusted to pH 5.6. To synchronize germination, sown seeds underwent stratification at 4 °C in complete darkness for a specified duration. For the 1-aminocyclopropane-1-carboxylic acid (ACC) treatment experiment, seeds were initially germinated in the dark on 1/2 MS medium for 7 days before being transferred to either fresh 1/2 MS medium or 1/2 MS medium supplemented with 10 μM ACC, followed by a 4.5 h incubation period prior to harvesting.

### EMS mutagenesis and suppressor screening

For EMS treatment, EMS solution was prepared by adding ddH_2_O to a 50 mL centrifuge tube up to a final volume of 50 mL, followed by the addition of 200 µL EMS (final concentration 0.4%). Approximately 50,000 *ctr1–1* seeds were incubated in the EMS solution at 25 °C for 8 h with shaking at 120 rpm. The tube was sealed and wrapped with aluminum foil to protect from light. After incubation, the EMS solution was discarded, and the seeds were washed 10 times with 40–50 mL ddH_2_O each time. The seeds were subsequently filtered through a 200–400 mesh nylon sieve and rinsed under running tap water for 4 h. Excess water was removed by placing the seeds on filter paper. The seeds were then spread on multiple layers of absorbent paper and newspaper in a fume hood and allowed to dry overnight at ambient temperature. The following day, the seeds were collected into a clean centrifuge tube containing silica gel desiccant and dried for 2–3 days. Subsequent to the drying process, the seeds were subjected to a dormancy-breaking treatment and sown in large trays (800–1000 seeds per tray). Seeds from each individual tray were harvested in bulk to generate the M2 population. For mutant screening, M2 seeds were directly sown in trays, and seedlings displaying significantly taller growth compared to the *ctr1–1* mutant were isolated as putative suppressors for further identification. Genetic mapping of the causative mutations was primarily achieved through whole-genome resequencing.

### Split luciferase complementation assay

The full-length *DCP1* gene and its mutant *DCP1m* (with a methionine-to-isoleucine substitution at position 78 of *DCP1*) were cloned into the *pCAMBIA-CLUC* vector, yielding recombinant plasmids *DCP1-cluc* and *DCP1-M78I-cluc*. The full-length *DCP2* gene and its mutant *DCP2m* (with an arginine-to-histidine substitution at position 138 of *DCP2*) were cloned into the *pCAMBIA-NLUC* vector, generating *DCP2-nluc* and *DCP2-R138H-nluc*. The C-terminal domain of *EIN2* (*EIN2C*) was cloned into both *pCAMBIA-NLUC* and *pCAMBIA-CLUC* vectors, resulting in *EIN2C-nluc* and *EIN2C-cluc*. All recombinant plasmids were subjected to restriction enzyme digestion and Sanger sequencing to confirm sequence accuracy and insert orientation. The verified plasmids were individually transformed into *Agrobacterium tumefaciens* strain *GV3101* via electroporation or freeze-thaw method. Positive clones were screened on selective media and validated by colony PCR using vector-specific primers. *Agrobacterium* suspensions were infiltrated into the abaxial epidermis of *Nicotiana benthamiana* leaves using a needleless syringe. Each plant was infiltrated in 2–3 leaves, with 50–100 μL of suspension per leaf. Two days post-infiltration, infiltrated leaves were excised and incubated in the dark with 1 mM D-Luciferin (in water) for 5–10 minutes. Luciferase activity was visualized using a NightShade LB 985 (Berthold) *in vivo* Plant Imaging System with a CCD camera, and bioluminescence intensity was quantified as relative light units using image analysis software.

### Bimolecular fluorescence complementation assay

Full-length *DCP1*, *DCP1m*, *DCP2*, *DCP2m*, *EIN2-C*, and *EIN5* were separately cloned into *pUC35S-nYFP* or *pUC35S-cYFP* (BIOGLE GeneTech) vectors, yielding *DCP2-cYFP*, *DCP2-R138H-cYFP*, *DCP1-nYFP*, *DCP1-M78I-nYFP*, *EIN5-cYFP*, *EIN5-nYFP* as well as *EIN2C-cYFP* and *EIN2C-nYFP*. These plasmids were co-transformed pairwise into *Arabidopsis* leaf protoplasts. The cells were then incubated in W5 solution for 10–12 hours to ensure sufficient expression of the fusion proteins. Finally, the expression of YFP (yellow fluorescent protein) was detected using a confocal laser scanning microscope (Zeiss).

### Co-IP assay

The full-length *DCP2* gene and C-terminal domain of *EIN2* (*EIN2C*) were cloned into the *Pcambia2300-GFP* vector respectively, yielding recombinant plasmids *DCP2-GFP* and *EIN2C -GFP*. The full-length *DCP1* gene were cloned into the *pCAMBIA-CLUC* vector, yielding recombinant plasmids *DCP1-cluc*. The C-terminal domain of *EIN2* (*EIN2C*) was cloned into *pCAMBIA-CLUC* vectors, resulting in *EIN2C-cluc*. All recombinant plasmids were verified through restriction enzyme digestion and Sanger sequencing to ensure correct insert sequence and orientation. The confirmed plasmids were subsequently introduced into *Agrobacterium tumefaciens* strain *GV3101* using either electroporation or freeze-thaw transformation methods. For transient expression, bacterial suspensions were infiltrated into the abaxial side of *Nicotiana benthamiana* leaves employing a needleless syringe, with each plant receiving injections in 2–3 leaves. At 48 hours post-infiltration, the infiltrated leaves were harvested and ground into fine powder under liquid nitrogen. The powdered tissues were lysed in RIPA buffer at 4 °C for 1.5 h, followed by centrifugation to collect the supernatant. After centrifugation at 13,000 g for 10 min, 20 μL of supernatant was reserved as input control, while the remainder was incubated with 30 μL of anti-GFP magnetic beads for 2 h at 4 °C . The beads were then washed five times with TBS buffer (50 mM Tris–HCl, pH 7.4, 150 mM NaCl, 0.1% Triton X-100), and the bound proteins were eluted using 70 μL of 2 × SDS loading buffer by heating at 95 °C for 5 min. The expressed LUC- and GFP-fused proteins were ultimately detected with anti-LUC (1:5,000; Sigma) and anti-GFP (1:5,000; ABclonal) antibodies via Western blot.

### Immunoblot assay

10-day-old seedlings of *35S::EIN3-3×FLAG/WT*, *35S::EIN3-3×FLAG/ems84* and *35S::EIN3-3×FLAG/ems64* were separately transferred to 1/2 MS solid medium in the presence and absence of 10 μM ACC treatment for 12 hours and 24 hours. After treatment, samples were collected and ground into powder in liquid nitrogen. The samples were lysed in RIPA buffer at 4 °C for 1.5 hours, followed by centrifugation to collect the supernatant. The supernatant was then boiled for 5 min at 95 °C in 2 × SDS sample buffer. Proteins were separated by SDS-PAGE, and the total proteins were detected using an α-Flag antibody (1:5000; ABclonal).

### *In vitro* decapping assay

Full-length *DCP1*, *DCP1M*, *DCP2* and *DCP2M* were individually cloned into *pCold-TF* and *PGEX4T-1* vectors. These plasmids were then transformed into *E. coli BL21 CodonPlus (DE3)* strains. The bacterial cultures were grown at 37 °C until they reached an optical density at 600 nm (OD600) of 0.6. Subsequently, protein expression was induced with 1 mM isopropyl β-D-1-thiogalactopyranoside (IPTG) at 16 °C for 16 h. The His-tagged recombinant proteins were purified with Ni-NTA Beads (Smart-Lifesciences, SA004025) and eluted using 2–3 ml of elution buffer (300 mM imidazole). The GST-tagged recombinant proteins were purified with Glutathione Beads (Smart-Lifesciences, SA008025) and eluted using 3 ml of elution buffer (15 mM reduced glutathione). Ultimately, purified proteins were aliquoted and stored at -80 °C until further use.

The purified proteins were incubated with capped mRNA (EZ Cap Firefly Luciferase mRNA) at 37 °C for 30 min. The decapping buffer was composed of 10 mM Tris-HCl (pH 7.5), 100 mM potassium acetate (KOAc), 2 mM magnesium acetate (MgOAc2), 0.5 mM manganese chloride (MnCl2), 2 mM dithiothreitol (DTT), 0.1 mM spermine, 25 μg/ml yeast tRNA, and an RNase inhibitor. Each sample was loaded into the wells and subjected to electrophoresis at 150 V for 30 minutes. The RNA was then transferred to a nitrocellulose membrane using a constant current of 380 mA for about 50 minutes. The RNA was detected using an Anti-7-Methylguanosine antibody (Abcam).

### RNA extraction and real-time quantitative PCR

Total RNA was extracted using the QIAzol reagent, adhering strictly to the manufacturer’s instructions provided by QIAGEN. The quality and integrity of the isolated RNA were evaluated by agarose gel electrophoresis. Subsequently, an equal amount of total RNA was subjected to reverse transcription with ABScript III RT Mix (ABclonal), following the well-established protocols detailed in previous studies. RT-qPCR analysis was performed using the SYBR Green Realtime PCR Master Mix (TOYOBO) on the Roche LightCycler480 real-time qPCR platform. The expression levels of individual mRNAs were quantified and normalized relative to the expression level of *ACTIN2* mRNAs using the *ΔCt* method.

### RNA-seq assay

Seeds were germinated and subsequently cultured in the dark on 1/2 MS plates supplemented with or without ACC for 5 days. Then rapid sampling was conducted for RNA extraction. The RNA-seq analysis was performed by Novogene Science and Technology Co. Ltd. (Tianjin, China) using the Illumina NovaSeq 6000 Sequencing System. Three biological replicates were performed. The differentially expressed genes were identified using edgeR (| log2Fold Change | > = 1, padj < 0.05). Raw data have been deposited to NCBI (accession: PRJNA1264772, PRJNA1444356 and PRJNA1263674).

### Statistical analysis

Univariate analysis of variance (ANOVA) was employed for the comparative analysis of strain indicators measured under a single condition, while two-way ANOVA was utilized for the comparison of indicators involving the interaction between two factors: experimental treatment and different strains. Statistical analyses were performed using GraphPad Prism 10.

## Data Availability

Raw data have been deposited to NCBI (accession: PRJNA1264772, PRJNA1444356 and PRJNA1263674).
