# Supplementary material for: mRNA decapping proteins regulate EIN2-dependent ethylene signaling in arabidopsis
Source: Front Plant Sci. 2026 Jun 30;17:1897707. doi: 10.3389/fpls.2026.1897707 (PMC13364847; doi:10.3389/fpls.2026.1897707)
Supplement: Supplementary file 3 [file Table3.docx]

Supplementary Material

**
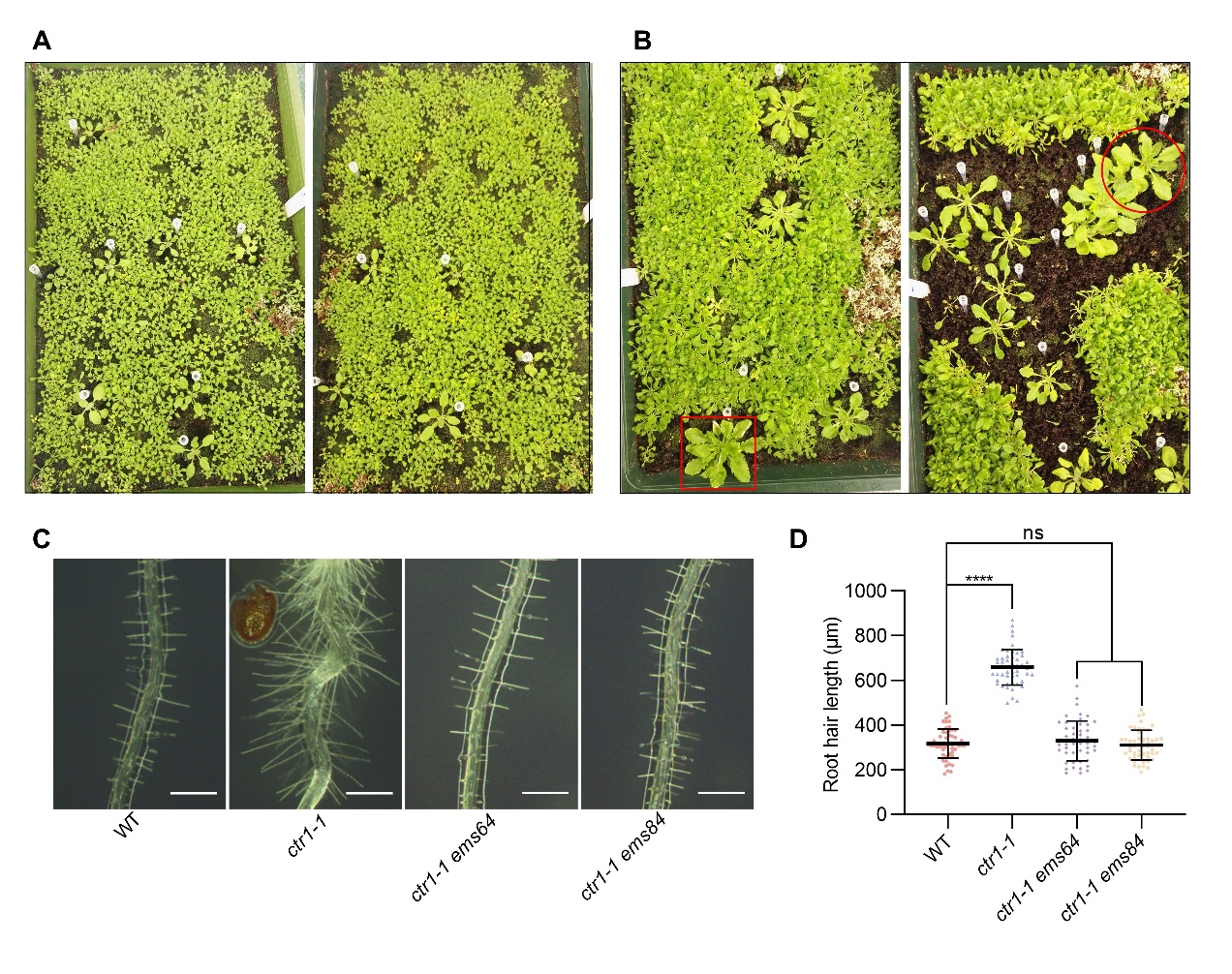
**

**Figure S1** Screening of *ctr1-1* suppressors. (A) Growth of 4-week-old *ctr1-1* *ems* mutants on soil. (B) Growth of 6-week-old *ctr1-1* *ems* mutants on soil. The one in the red square indicates *ctr1-1 ems64*, and the one in the red circle indicates *ctr1-1 ems84*. (C) Images of root hairs of WT, *ctr1-1*, *ctr1-1 ems64* and *ctr1-1 ems84* in 7-day-old seedlings. Scale bars, 500 μm. (D) Quantification of root hair length in (C) (50 root hairs from 5 roots of each line were measured). Data means ± SD, unpaired t test in (D) (*****P* < 0.0001, ns means not significant) were used.


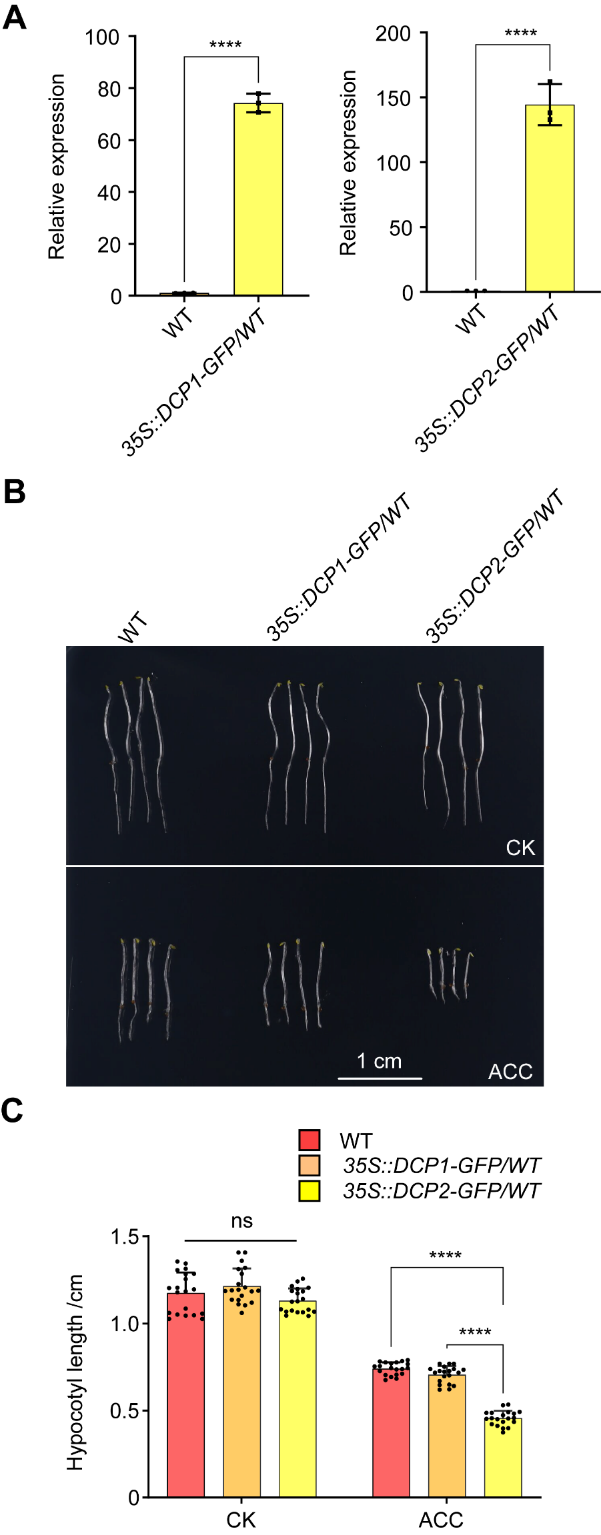


**Figure S2** Response of *DCP1/DCP2-GFP* overexpression lines to ACC treatment. (A) Expression of *DCP1* and *DCP2* in the overexpression lines. (B) Phenotypes and (C) hypocotyl length of *35S:DCP1-GFP* and *35S:DCP2-GFP* lines grown on ½ MS solid medium with or without 10 μM ACC treatment for 7 days under dark condition (n = 20). Data are shown as means ± SD. Data were analyzed with one-way ANOVA (‘ns’ means no significant differences, *****P* < 0.0001).


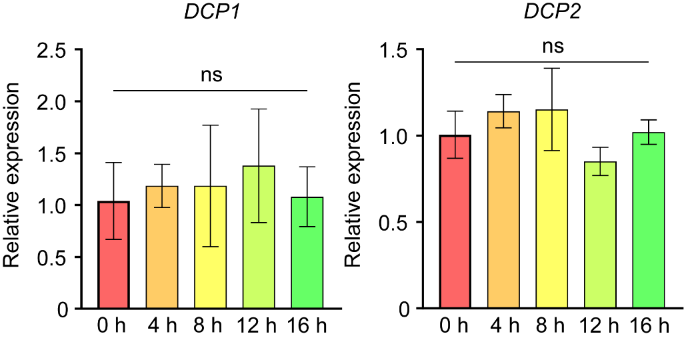


**Figure S3** Expression analysis of *DCP1*and *DCP2* in WT. Total RNAs were extracted from 7-day-old seedlings with 10 µM ACC for 0h, 4h, 8 h, 12 h, and 16 h, respectively (n = 3). Data are shown as means ± SD. Data were analyzed with one-way ANOVA (‘ns’ means no significant differences).

**
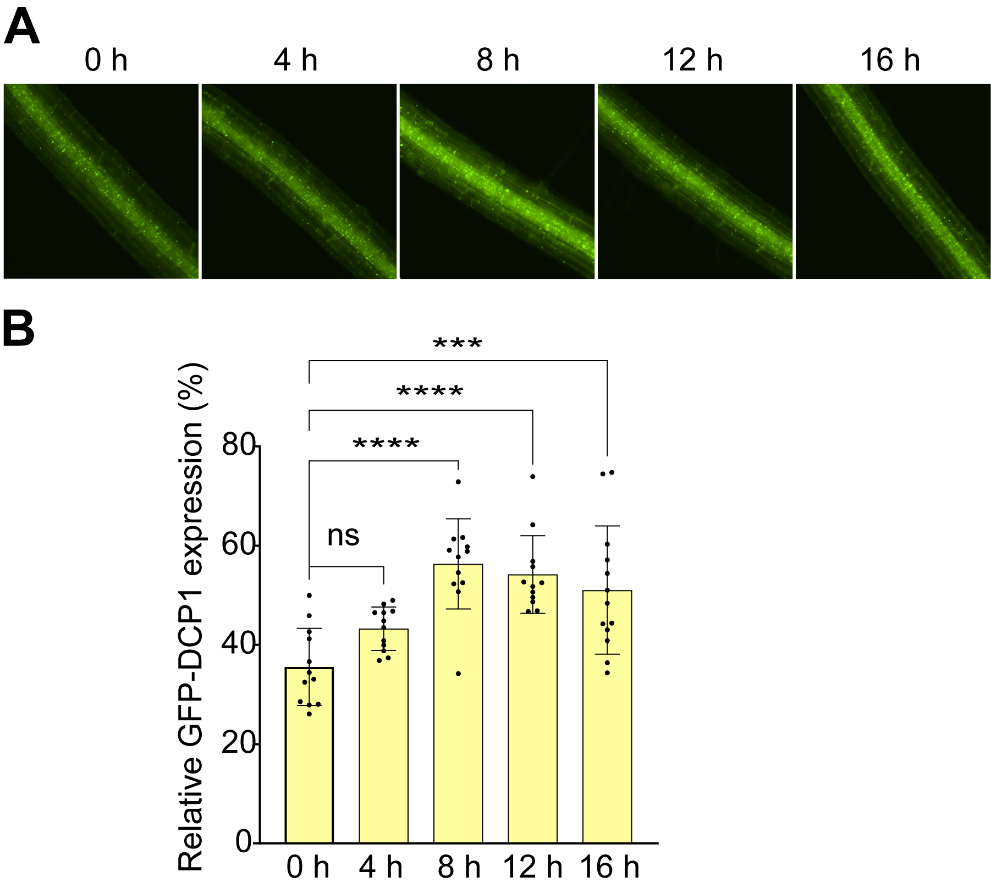
**

**Figure S4** GFP-DCP1 fluorescence signals in roots. (A) GFP-DCP1 fluorescence signals in roots with 10 µM ACC for 0h, 4h, 8 h, 12 h, and 16 h, respectively. (B) Relative quantification under ACC treatment (n = 12). Data are shown as means ± SD. Data in different time point was compared by Dunnett’s multiple comparisons test with the data in 0 h as the control. (‘ns’ means no significant differences; ****P* < 0.001, *****P* < 0.0001).


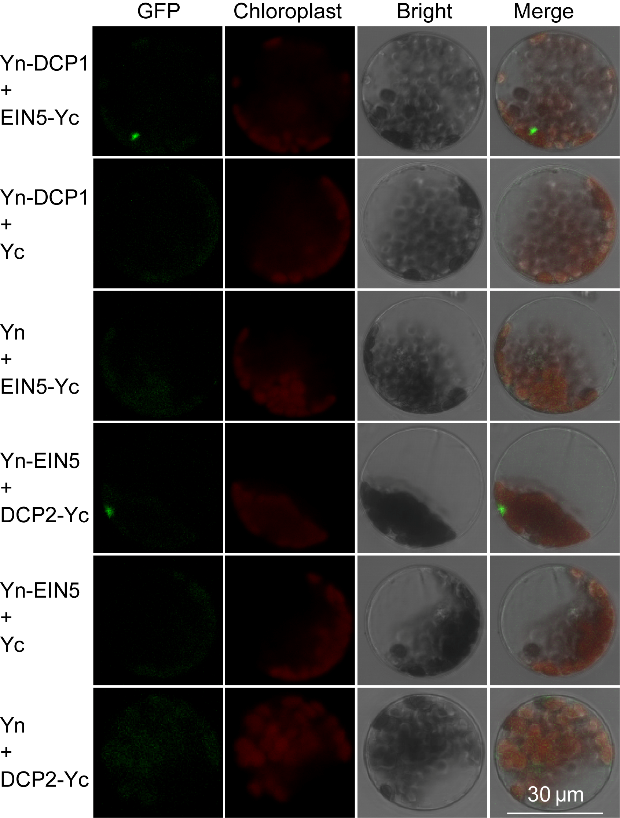


**Figure S5** BiFC showing the interaction of DCP1and DCP2 with EIN5.


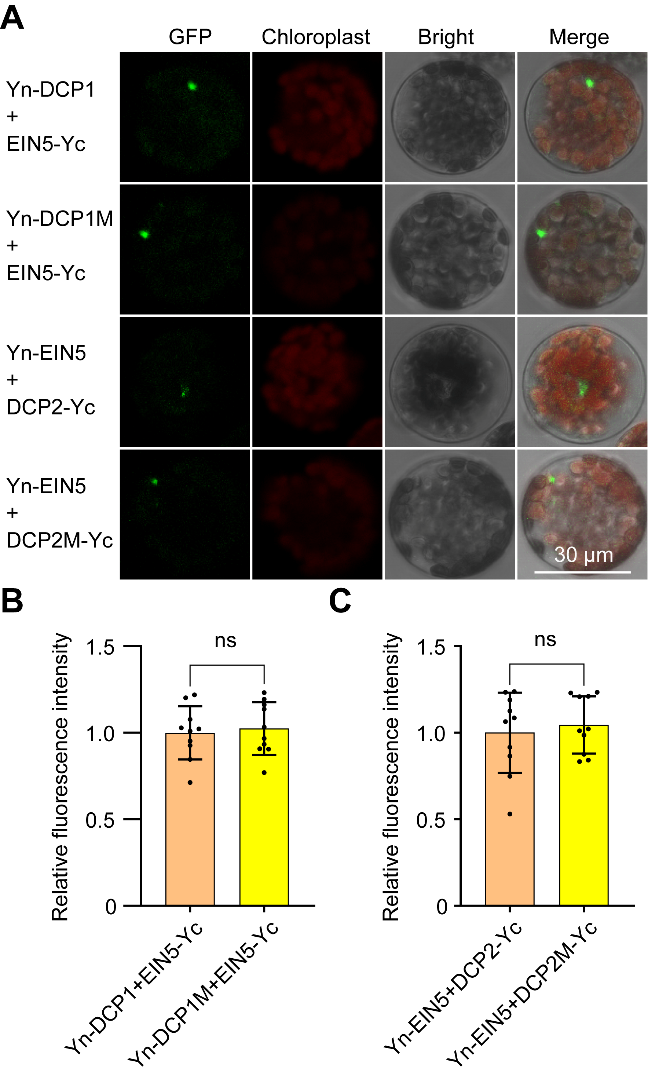


**Figure S6** Interaction between DCP1M/2M and EIN5. (A) BiFC showing that DCP1M/2M did not affect the interaction with EIN5. (B) The fluorescence intensity of BiFC in (A). Data are shown as means ± SD. Data were analyzed with unpaired t test (‘ns’ means no significant differences).


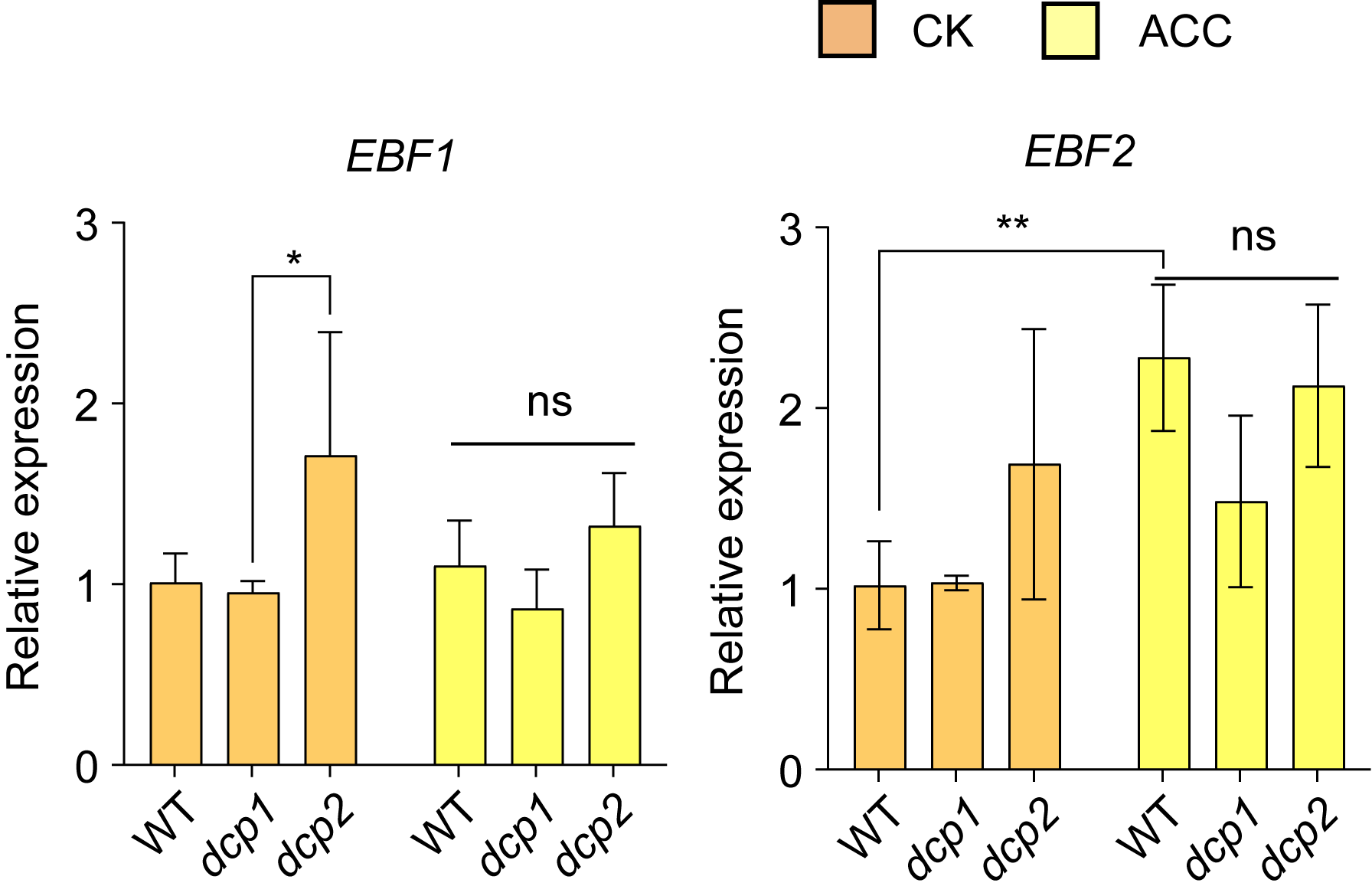


**Figure S7** Expression analysis of *EBF1*and *EBF2* in WT, *dcp1* and *dcp2*. Total RNAs were extracted from 5-day-old seedlings under control and ACC treatment for 4.5 hrs (n = 3). Data are shown as means ± SD. Data were analyzed with one-way ANOVA (‘ns’ means no significant differences, *P < 0.05).


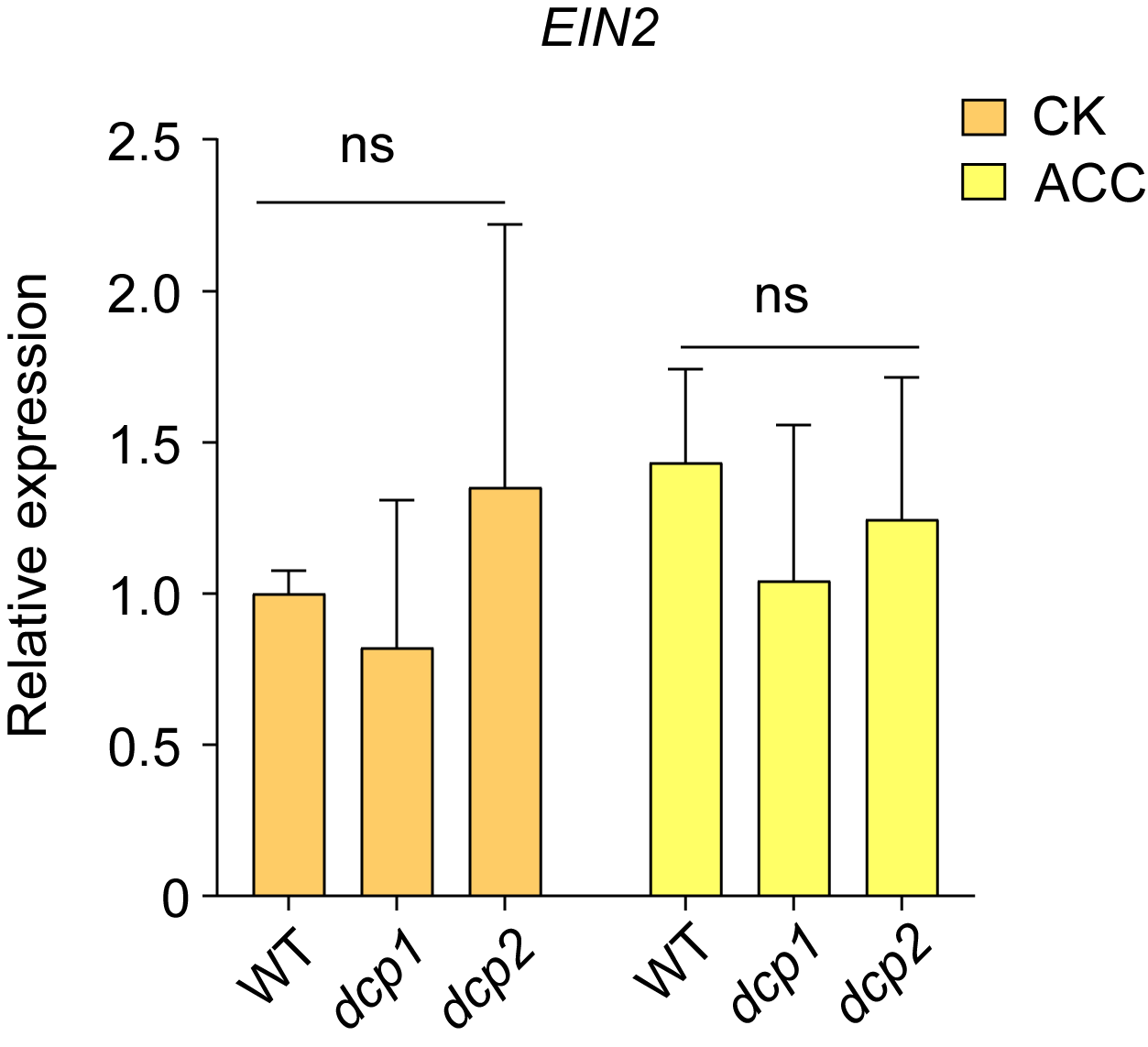


**Figure S8** Expression analysis of *EIN2* in WT, *dcp1* and *dcp2* under control and ACC treatment. Total RNAs were extracted from 7-day-old seedlings under control and ACC treatment for 4.5 hrs (n = 3). Data are shown as means ± SD. Data were analyzed with two-way ANOVA (‘ns’ means no significant differences).


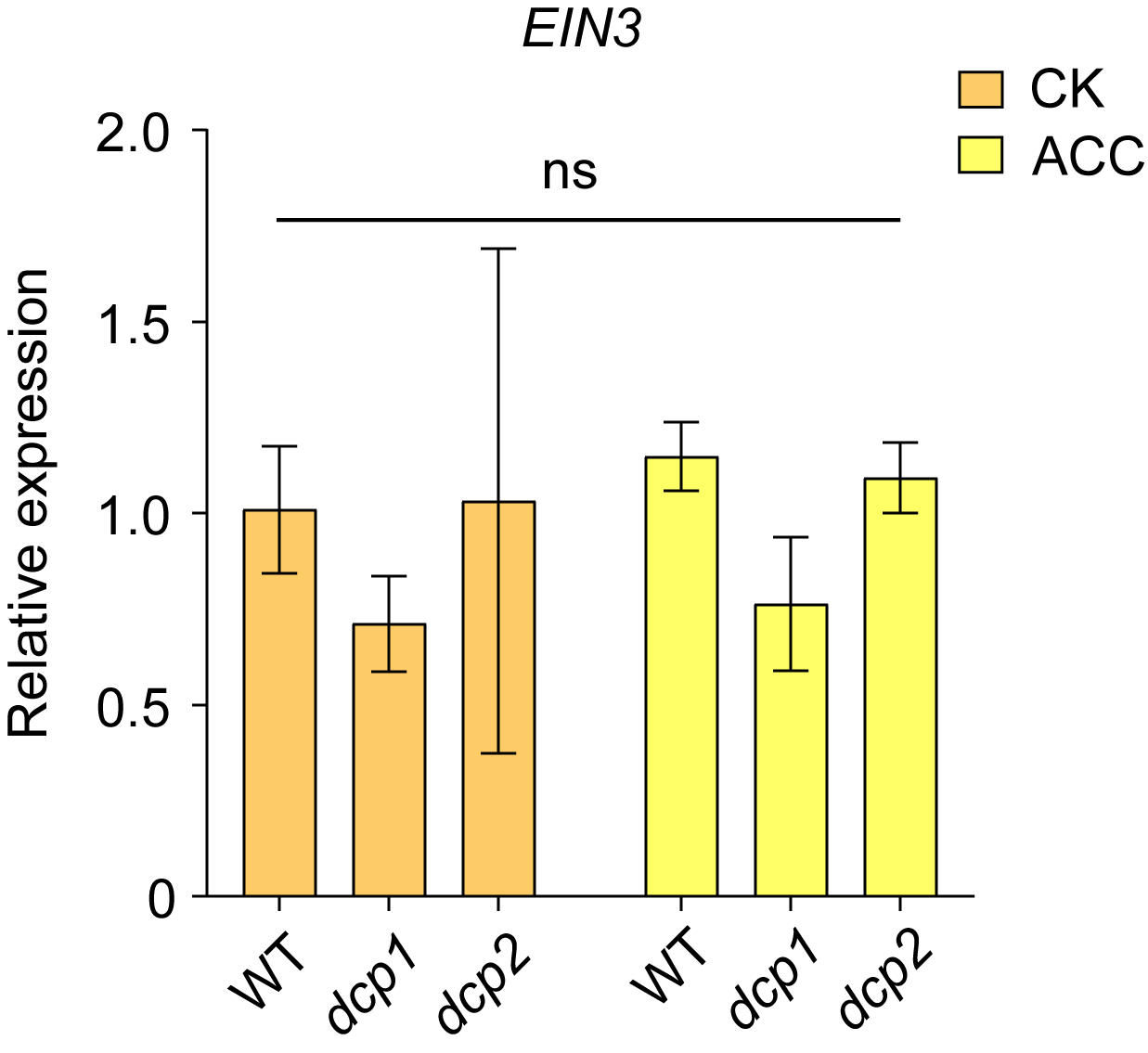


**Figure S9** The transcript levels of *EIN3* are not regulated by ethylene.

Expression analysis of *ERF1* in WT, *dcp1* and *dcp2* under control and ACC treatment. Total RNAs were extracted from 7-day-old seedlings under control and ACC treatment for 4.5 hrs (n = 3). Data are shown as means ± SD. Data were analyzed with two-way ANOVA (‘ns’ means no significant differences).


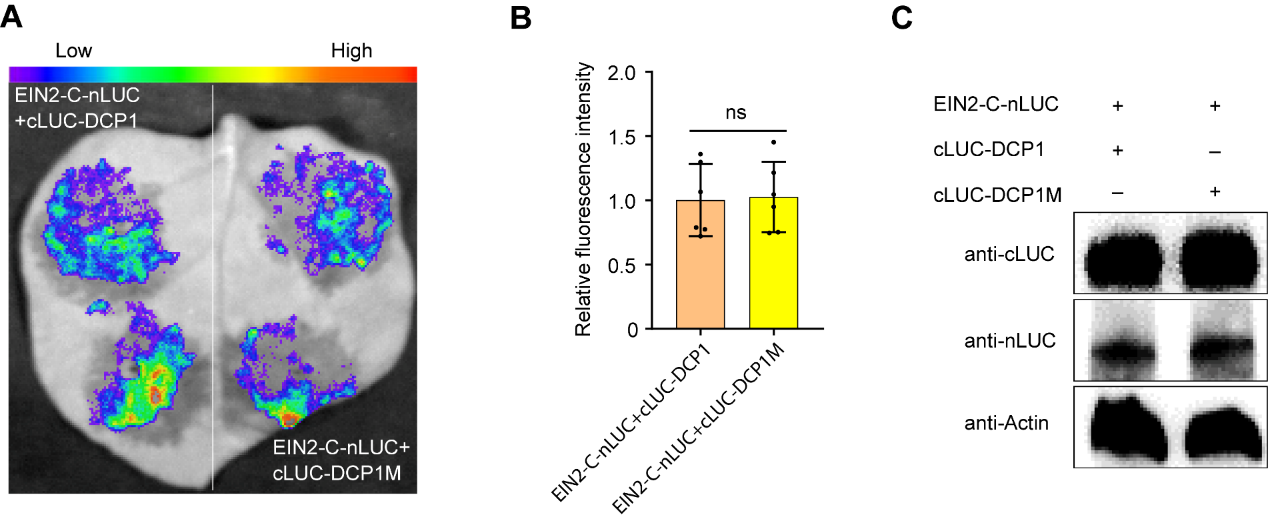


**Figure S10** Interaction between DCP1/DCP1M and EIN2-C. (A) Split-LUC assays showing the interaction of DCP1 and DCP1M with EIN2-C.（B）The luciferase intensity of LUC in (A). (C) Immunoblot analysis. Data are shown as means ± SD. Data were analyzed with t test (‘ns’ means no significant differences).


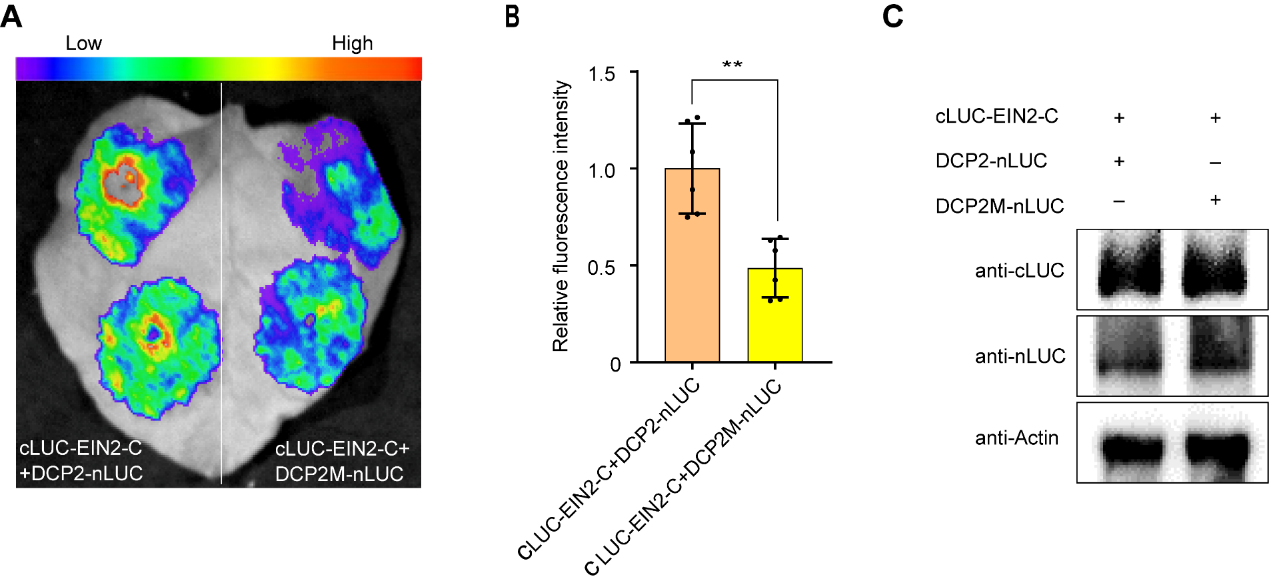


**Figure S11** Interaction between DCP2/DCP2M and EIN2-C. (A) Split-LUC assays showing the interaction of DCP2and DCP2M with EIN2-C. (B) The luciferase intensity of LUC in (A). (C) Immunoblot analysis. Data are shown as means ± SD. Data were analyzed with t test (***P* < 0.01).


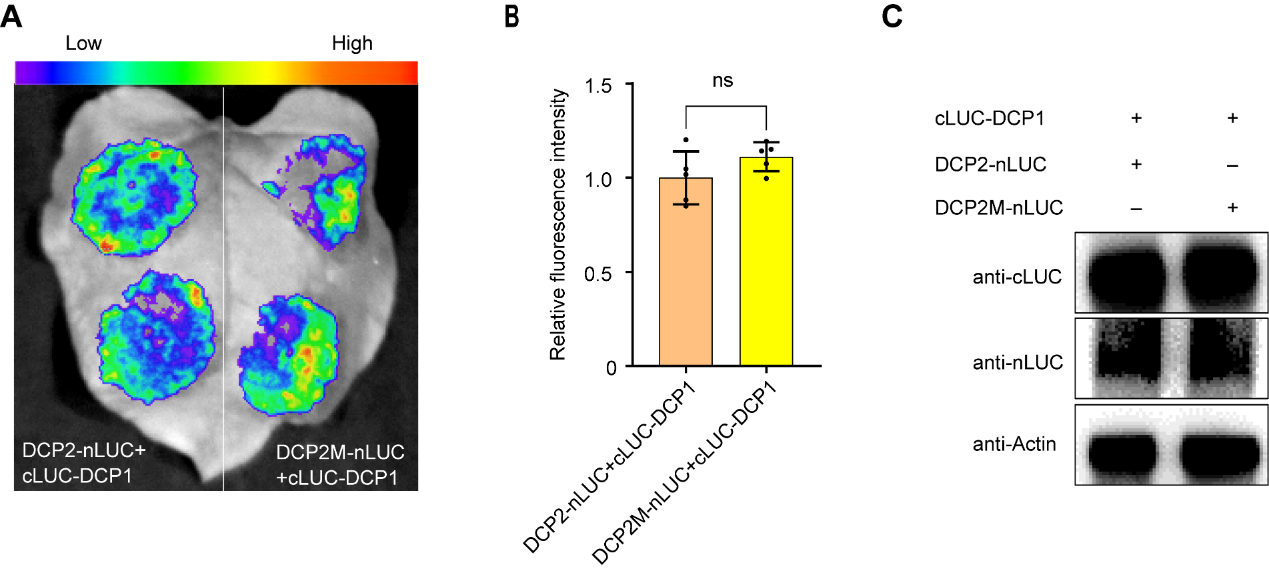


**Figure S12** Interaction between DCP2/DCP2M and DCP1. (A) Split-LUC assays showing the interaction of DCP1 and DCP1M with EIN2-C.（B）The luciferase intensity of LUC in (A). (C) Immunoblot analysis. Data are shown as means ± SD. Data were analyzed with t test (‘ns’ means no significant differences).


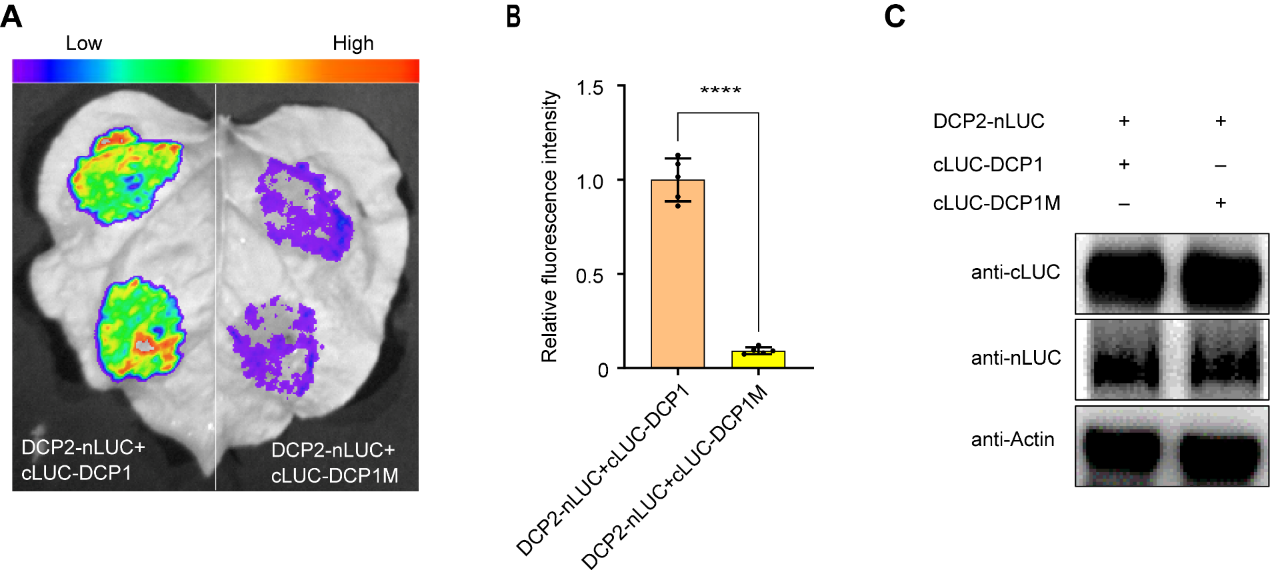


**Figure S13** Interaction between DCP1/DCP1M and DCP2. (A) Split-LUC assays showing the interaction of DCP1 and DCP1M with EIN2-C.（B）The luciferase intensity of LUC in (A). (C) Immunoblot analysis. Data are shown as means ± SD. Data were analyzed with t test (*****P* < 0.0001).


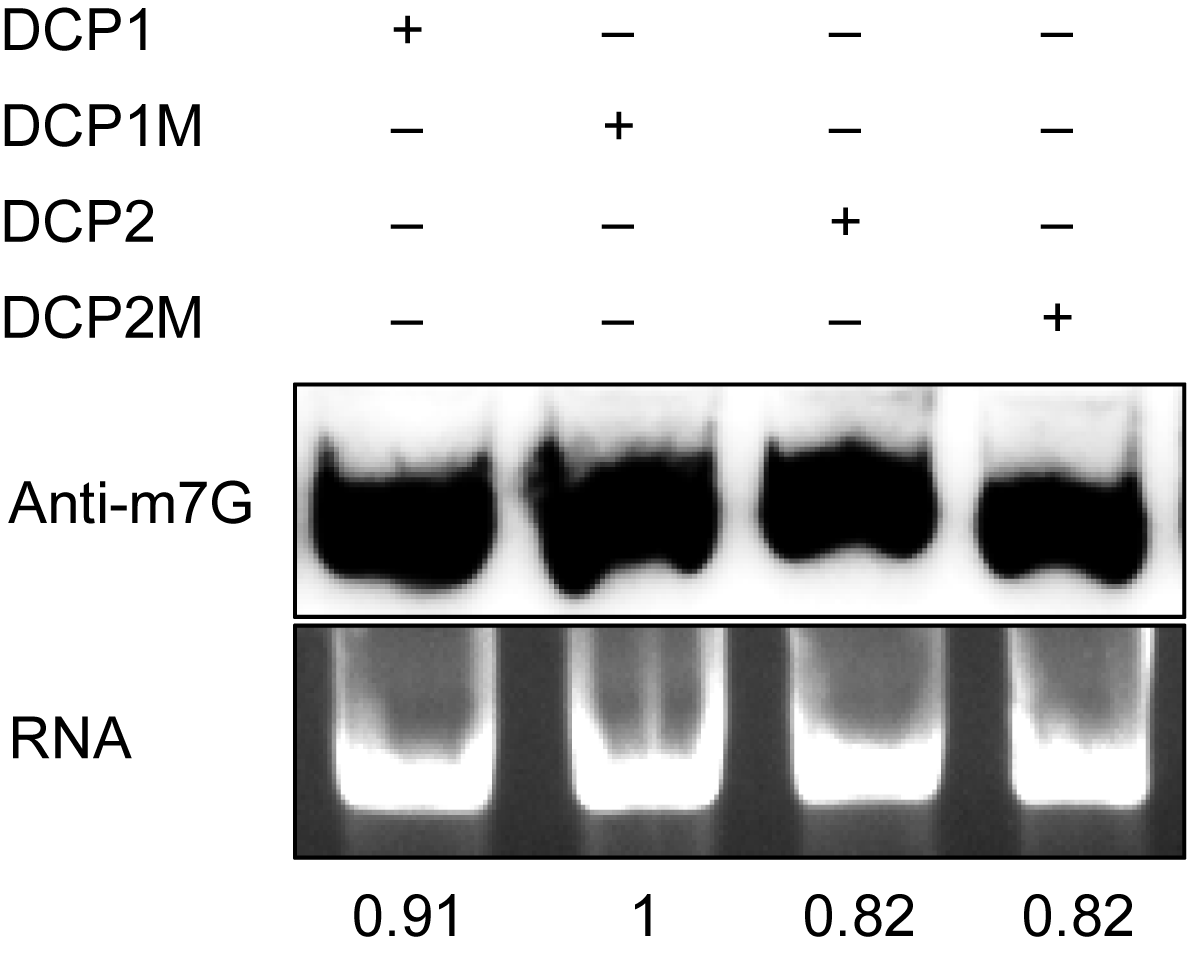


**Figure S14** EMS-induced mutations in DCP1/2 affect DCP decapping function. The mRNA used for the experiment was the EZ Cap™ Firefly Luciferase mRNA. The decapping activity of DCPs was assessed by monitoring the reduction of 5’-cap structures in mRNA. The cap structure was detected with Anti-7-Methylguanosine antibody. Detection of RNA in each analysis was used as the loading control.

**Table S1** Mutant genes in *ctr1-1EMS64* and *ctr1-1EMS84*

| **Gene** | **Mut** | **Mut** | **Position** | **Description** |
| --- | --- | --- | --- | --- |
| ***ctr1-1*EMS64** | | | | |
| gene:AT5G05850 | C | T | Chr5:1764213 | PIRL1 |
| gene:AT5G06140 | C | T | Chr5:1858365 | SNX1 |
| gene:AT5G06220 | C | T | Chr5:1882702 | LETM1-like protein |
| gene:AT5G06230 | C | T | Chr5:1885529 | TBL9 |
| gene:AT5G07940 | G | A | Chr5:2539509 | SYS1 |
| gene:AT5G08230 | G | A | Chr5:2647925 | HULK1 |
| gene:AT5G10200 | G | A | Chr5:3203020 | SAV4 |
| gene:AT5G11610 | G | A | Chr5:3736772 | Exostosin family protein |
| gene:AT5G11620 | G | A | Chr5:3739974 | SWIM zinc finger family protein |
| gene:AT5G13030 | G | A | Chr5:4134628 | selenoprotein O |
| gene:AT5G13570 | G | A | Chr5:4368326 | DCP2 |
| gene:AT5G18410 | C | T | Chr5:6101830 | PIR121 |
| ***ctr1-1*EMS84** | | | | |
| gene:AT1G01110 | G | A | Chr1:53824 | IQ-Domain 18 |
| gene:AT1G07650 | G | A | Chr1:2364654 | LMK1 |
| gene:AT1G07720 | G | A | Chr1:2392114 | KCS3 |
| gene:AT1G08300 | G | A | Chr1:2617827 | No vein-like protein |
| gene:AT1G08350 | G | A | Chr1:2633676 | Endomembrane protein 70 protein family |
| gene:AT1G08370 | G | A | Chr1:2638755 | DCP1 |

| **Table S2** Sequence of primers used in this study | |
| --- | --- |
| **Name** | **Nucleotide Sequence (5’ to 3’)** |
| **Characterization of mutants** | |
| DCP2-F | GCCACATAGACTGGGCTTCTCTTTGTCTTTTC |
| DCP2-R | CTCTGTTTCAAGAGCAGAAGATATAAGAGACG |
| DCP1-F | ATGTCTCAAAACGGGAAGATAATCCCAAAT |
| DCP1-R | GCCAAGGAACCATTAGGCAGAGATACAACG |
| CTR1-F | CGTCGCCTGAGTATGGCTTAT |
| CTR1-R | TTAGACGGCTCATCTCGCAG |
| EIN5-F | TCTGATTGAAATGGGAAAACG |
| EIN5-R | CCTTTCCTTCAGGATGGAAAC |
| **Primers for qRT-PCR** | |
| ACTIN2/8-F | GGTAACATTGTGCTCAGTGGTGG |
| ACTIN2/8-R | AACGACCTTAATCTTCATGCTGC |
| Qsl-ERF1-F | ATTAGGGTTTGGCTCGGGAC |
| Qsl-ERF1-R | CTCTTGAACTCTCTCCGCCG |
| Qsl-HLS1-F | CTCTGTTCTTTACTTATACCTCACG |
| Qsl-HLS1-R | TCGAGAGGAAAGAGAAGCATTT |
| Qsl-RAP2.2-F | TCGGCTTTTGACTGCGAAGA |
| Qsl-RAP2.2-R | ACCAAATATATACCAGTGGAGACGA |
| Qsl-ERF12-F | CACCTCCTCGCCTAACACAG |
| Qsl-ERF12-R | CTCACCGTAACAGGGGAAGG |
| Qsl-DCP1-F | CAGCGATTCCTCACCAACCT |
| Qsl-DCP1-R | AGGAGGATTGTAGAGGCGGT |
| Qsl-DCP2-F | TGAAATCACATGGCATCGGC |
| Qsl-DCP2-R | CCACGACTTCAATGACGAAAGG |
| Qsl-EBF1-F | TGATCTGTGTGGGCTCAAGG |
| Qsl-EBF1-R | CGAGCAGTGATGGCAGAGAT |
| Qsl-EBF2-F | TTCGTGGGAGTGGGTTTGAG |
| Qsl-EBF2-R | GACCGTGCGATCTCAGACAA |
| Qsl-EIN3-F | GGTCCTGCGGCTATTACCAA |
| Qsl-EIN3-R | AGCGATCCAAGAGTCGTGTC |
| Qsl-EIN2-F | GTGGAGGAGGAGGGTATGGT |
| Qsl-EIN2-R | CCACCCGTGACCCCAAATTA |
| Qsl-EIN3-3XFlag-F | CATTTCATTTGGAGAGAACACG |
| Qsl-EIN3-3XFlag -R | CCCTCGACTTTATCGTCA |
| **Primers for transgenic plants constructs** | |
| 35S::EIN3-3×Flag-F | AAAGTCGAGGGGGGGCCCGGTACCATGATGTTTAATGAGATGG |
| 35S::EIN3-3×Flag-R | TACTCGAACCTGCAGGTCGACTTAGAACCATATGGATACATC |
| 35S:DCP2-GFP-1301-F | CACGGGGGACGGTACCCGGGGATCCATGTCGGGCCTCCATCGATC |
| 35S:DCP2-GFP-1301-R | TGCTCACCATTCCTGGGTCGTCGACAGCTGAATTACCAGATTCCAACGC |
| 35S:DCP1-GFP-1301-F | CACGGGGGACGGTACCCGGGGATCCATGTCTCAAAACGGGAAGATA |
| 35S:DCP1-GFP-1301-R | TGCTCACCATTCCTGGGTCGTCGACTTGTTGAAGTGCATTTTGTA |
| **Primers for BiFC constructs** | |
| DCP1-nYn -F | TTCTGAGGAGGATCTTAGGCCTATGTCTCAAAACGGGAAGATAATCC |
| DCP1-nYn-R | CAAATGTTTGAACGATCTGCAGTCATTGTTGAAGTGCATTTTGTAAAG |
| DCP2-cYc-F | TCTCCCCTTGCTCCGTGGATCCATGTCGGGCCTCCATCGATCATCAAGTTC |
| DCP2-cYc-R | TTAGCGTGTGAAGAGCAGGCCTAGCTGAATTACCAGATTCCAACGCCTG |
| EIN5-nYn -F | TTCTGAGGAGGATCTTAGGCCTATGGGAGTACCGGCGTTCTACAGATGGC |
| EIN5-nYn -R | CAAATGTTTGAACGATCCCGGGTCACAAGTTTGCACCTCGATGACTTGGTTGTG |
| EIN2-nYn -F | TTCTGAGGAGGATCTTAGGCCT ATGGAAGCTGAAATTGTGAATGTGAG |
| EIN2-nYn -R | CAAATGTTTGAACGATCTGCAGTCAACCCAATGATCCGTACGCAGTCACG |
| EIN2-cYc-F | TCTCCCCTTGCTCCGTGGATCC ATGGAAGCTGAAATTGTGAATGTGAG |
| EIN2-cYc-R | TTAGCGTGTGAAGAGCAGGCCTACCCAATGATCCGTACGCAGTCACG |
| **Primers for split-LUC Complementation constructs** | |
| EIN2-nluc-F | AGAGAACACGGGGGACGAGCTTATGGAAGCTGAAATTGTGAATGTGAG |
| EIN2-nluc-R | CCCGGGACGCGTACGAGATCTGACCCAATGATCCGTACGCAGTCACG |
| EIN2-cluc-F | GTACGCGTCCCGGGGCGGTACCATGGAAGCTGAAATTGTGAATGTGAG |
| EIN2-cluc-R | AACGAAAGCTCTGCAGGTCGACTCAACCCAATGATCCGTACGCAGTC |
| DCP1-cluc-F | GTACGCGTCCCGGGGCGGTACCATGTCTCAAAACGGGAAGATAATCC |
| DCP1-cluc-R | AACGAAAGCTCTGCAGGTCGACTCATTGTTGAAGTGCATTTTGTAAAG |
| DCP2-cluc-F | GTACGCGTCCCGGGGCGGTACCATGTCGGGCCTCCATCGATCATCAAGT |
| DCP2-cluc-R | AACGAAAGCTCTGCAGGTCGACTCAAGCTGAATTACCAGATTCCAACGC |
| DCP1-nluc-F | AGAGAACACGGGGGACGAGCTTATGTCTCAAAACGGGAAGATAATCC |
| DCP1-nluc-R | CCCGGGACGCGTACGAGATCTGTTGTTGAAGTGCATTTTGTAAAG |
| DCP2-nluc-F | AGAGAACACGGGGGACGAGCTTATGTCGGGCCTCCATCGATCATCAAGT |
| DCP2-nluc-R | CCCGGGACGCGTACGAGATCTGAGCTGAATTACCAGATTCCAACGC |
| **Primers for recombinant protein constructs** | |
| DCP2-Pcold-His-F | GAAGGTAGGCATATGGAGCTCGGTACCATGTCGGGCCTCCATCGATC |
| DCP2-Pcold-His-R | TACCTATCTAGACTGCAG GTCGACTCAAGCTGAATTACCAGATT |
| 35s-DCP2-2300-1-F | GGGGGACGAGCTCGGTACCATGTCGGGCCTCCATCGATCATCAAGT |
| 35s-DCP2-2300-1-R | TTGTCGACTCTAGAGGATCCAGCTGAATTACCAGATTCCAACGC |
| 35s-EIN2C-2300-1-F | GGGGGACGAGCTCGGTACCATGAAATCTGCGAGTAACAGAGCGGAAGC |
| 35s-EIN2C-2300-1-R | TTGTCGACTCTAGAGGATCCACCCAATGATCCGTACGCAGTCACGTTTT |
| DCP1-GST-F | CTGGTTCCGCGTGGATCCATGTCTCAAAACGGGAAGAT |
| DCP1-GST-R | CTCGAGTCGACCCGGGAATTCTCATTGTTGAAGTGCATTTT |
|  |  |
